# Supplementary material for: tRNA as an assembly chaperone for a macromolecular transcription-processing complex
Source: Nat Struct Mol Biol. 2025 Sep 4;32(11):2349–58. doi: 10.1038/s41594-025-01653-y (PMC12618233; doi:10.1038/s41594-025-01653-y)
Supplement: Supplementary file 1 — Supplementary Table 1: Alignment of relevant tRNAGln isodecoders and their isoforms. [file 41594_2025_1653_MOESM1_ESM.pdf]

nature structural & molecular biology

Supplementary information https://doi.org/10.1038/s41594-025-01653-y

tRNA as an assembly chaperone for a macromolecular transcription-processing complex

In the format provided by the authors and unedited

```
Anticodon                                          XXX
Gln-TTG-1-1       GGUCCCAUGGUGUAAUGGUUAGCACUCUGGACUUUGAAUCCAGCGA-UCCGAGUUCAAAU
Gln-CTG-6-1       GGCCCCAUGGUGUAAUGGUCAGCACUCUGGACUCUGAAUCCAGCGA-UCCGAGUUCAAAU
Gln-TTG-3-1       GGCCCCAUGGUGUAAUGGUUAGCACUCUGGACUUUGAAUCCAGCGA-UCCGAGUUCAAAU
Gln-TTG-3-2       GGCCCCAUGGUGUAAUGGUUAGCACUCUGGACUUUGAAUCCAGCGA-UCCGAGUUCAAAU
Gln-TTG-3-3       GGCCCCAUGGUGUAAUGGUUAGCACUCUGGACUUUGAAUCCAGCGA-UCCGAGUUCAAAU
Gln-TTG-4-1       GGUCCCAUGGUGUAAUGGUUAGCACUCUGGGCUUUGAAUCCAGCAA-UCCGAGUUCGAAU
Gln-TTG-2-1       GGUCCCAUGGUGUAAUGGUUAGCACUCUGGACUUUGAAUCCAGCAA-UCCGAGUUCGAAU
Gln-CTG-5-1       GGUUCCAUGGUGUAAUGGUUAGCACUCUGGACUCUGAAUCCGGUAA-UCCGAGUUCAAAU
Gln-CTG-7-1       GGUUCCAUGGUGUAAUGGUAAGCACUCUGGACUCUGAAUCCAGCCA-UCUGAGUUCGAGU
Gln-CTG-3-1       GGUUCCAUGGUGUAAUGGUGAGCACUCUGGACUCUGAAUCCAGCGA-UCCGAGUUCGAGU
Gln-CTG-3-2       GGUUCCAUGGUGUAAUGGUGAGCACUCUGGACUCUGAAUCCAGCGA-UCCGAGUUCGAGU
Gln-CTG-4-1       GGUUCCAUGGUGUAAUGGUAAGCACUCUGGACUCUGAAUCCAGCGA-UCCGAGUUCGAGU
Gln-CTG-4-2       GGUUCCAUGGUGUAAUGGUAAGCACUCUGGACUCUGAAUCCAGCGA-UCCGAGUUCGAGU
Gln-CTG-1-1       GGUUCCAUGGUGUAAUGGUUAGCACUCUGGACUCUGAAUCCAGCGA-UCCGAGUUCAAAU
Gln-CTG-1-2       GGUUCCAUGGUGUAAUGGUUAGCACUCUGGACUCUGAAUCCAGCGA-UCCGAGUUCAAAU
Gln-CTG-1-3       GGUUCCAUGGUGUAAUGGUUAGCACUCUGGACUCUGAAUCCAGCGA-UCCGAGUUCAAAU
Gln-CTG-1-4       GGUUCCAUGGUGUAAUGGUUAGCACUCUGGACUCUGAAUCCAGCGA-UCCGAGUUCAAAU
Gln-CTG-1-5       GGUUCCAUGGUGUAAUGGUUAGCACUCUGGACUCUGAAUCCAGCGA-UCCGAGUUCAAAU
Gln-CTG-2-1       GGUUCCAUGGUGUAAUGGUUAGCACUCUGGACUCUGAAUCCAGCGA-UCCGAGUUCAAGU
Arg-CCG-2-1       GACCCAGUGGCCUAAUGGAUAAGGCAUCAGCCUCCGGAGCUGGGGAUUGUGGGUUCGAGU
Arg-ACG-1-1       GGGCCAGUGGCGCAAUGGAUAACGCGUCUGACUACGGAUCAGAAGAUUCCAGGUUCGACU
Arg-ACG-1-2       GGGCCAGUGGCGCAAUGGAUAACGCGUCUGACUACGGAUCAGAAGAUUCCAGGUUCGACU
Arg-ACG-1-3       GGGCCAGUGGCGCAAUGGAUAACGCGUCUGACUACGGAUCAGAAGAUUCCAGGUUCGACU
Arg-ACG-2-1       GGGCCAGUGGCGCAAUGGAUAACGCGUCUGACUACGGAUCAGAAGAUUCUAGGUUCGACU
Arg-ACG-2-2       GGGCCAGUGGCGCAAUGGAUAACGCGUCUGACUACGGAUCAGAAGAUUCUAGGUUCGACU
Arg-ACG-2-3       GGGCCAGUGGCGCAAUGGAUAACGCGUCUGACUACGGAUCAGAAGAUUCUAGGUUCGACU
Arg-ACG-2-4       GGGCCAGUGGCGCAAUGGAUAACGCGUCUGACUACGGAUCAGAAGAUUCUAGGUUCGACU
Arg-TCG-5-1       GACCACGUGGCCUAAUGGAUAAGGCGUCUGACUUCGGAUCAGAAGAUUGAGGGUUCGAAU
Arg-TCG-2-1       GACCACGUGGCCUAAUGGAUAAGGCGUCUGACUUCGGAUCAGAAGAUUGAGGGUUCGAAU
Arg-TCG-4-1       GACCACGUGGCCUAAUGGAUAAGGCGUCUGACUUCGGAUCAGAAGAUUGAGGGUUCGAAU
Arg-CCG-1-1       GGCCGCGUGGCCUAAUGGAUAAGGCGUCUGAUUCCGGAUCAGAAGAUUGAGGGUUCGAGU
Arg-CCG-1-2       GGCCGCGUGGCCUAAUGGAUAAGGCGUCUGAUUCCGGAUCAGAAGAUUGAGGGUUCGAGU
Arg-CCG-1-3       GGCCGCGUGGCCUAAUGGAUAAGGCGUCUGAUUCCGGAUCAGAAGAUUGAGGGUUCGAGU
Arg-TCG-3-1       GACCGCGUGGCCUAAUGGAUAAGGCGUCUGACUUCGGAUCAGAAGAUUGAGGGUUCGAGU
Arg-TCG-6-1       GGCCGUGUGGCCUAAUGGAUAAGGCGUCUGACUUCGGAUCAAAAGAUUGCAGGUUUGAGU
Arg-TCG-1-1       GGCCGCGUGGCCUAAUGGAUAAGGCGUCUGACUUCGGAUCAGAAGAUUGCAGGUUCGAGU
                  *      ***   *****  *   *    *  *  * * *     * *    ***  * *
```

Gln-TTG-1-1	CUCGGUGGGACCU
Gln-TTG-3-1	CUCGGUGGGACCU
Gln-TTG-3-2	CUCGGUGGGACCU
Gln-TTG-3-3	CUCGGUGGGACCU
Gln-TTG-4-1	CUUGGUGGGACCU
Gln-TTG-2-1	CUCGGUGGGACCU
Gln-CTG-5-1	CUCGGUGGAACCU
Gln-CTG-7-1	CUCUGUGGAACCU
Gln-CTG-3-1	CUCGGUGGAACCU
Gln-CTG-3-2	CUCGGUGGAACCU
Gln-CTG-4-1	CUCGGUGGAACCU
Gln-CTG-4-2	CUCGGUGGAACCU
Gln-CTG-1-1	CUCGGUGGAACCU
Gln-CTG-1-2	CUCGGUGGAACCU
Gln-CTG-1-3	CUCGGUGGAACCU
Gln-CTG-1-4	CUCGGUGGAACCU
Gln-CTG-1-5	CUCGGUGGAACCU
Gln-CTG-2-1	CUCGGUGGAACCU
Arg-CCG-2-1	CCCAUCUGGGUCG
Arg-ACG-1-1	CCUGGCUGGCUCG
Arg-ACG-1-2	CCUGGCUGGCUCG
Arg-ACG-1-3	CCUGGCUGGCUCG
Arg-ACG-2-1	CCUGGCUGGCUCG
Arg-ACG-2-2	CCUGGCUGGCUCG
Arg-ACG-2-3	CCUGGCUGGCUCG
Arg-ACG-2-4	CCUGGCUGGCUCG
Arg-TCG-5-1	CCCUUCGUGGUUG
Arg-TCG-2-1	CCCUCCGUGGUUA
Arg-TCG-4-1	CCCUUCGUGGUUA
Arg-CCG-1-1	CCCUUCGUGGUCG
Arg-CCG-1-2	CCCUUCGUGGUCG
Arg-CCG-1-3	CCCUUCGUGGUCG
Arg-TCG-3-1	CCCUUCGUGGUCG
Arg-TCG-6-1	UCUGCCACGGUCG
Arg-TCG-1-1	CCUGCCGCGGUCG
