# Supplementary material for: tRNA as an assembly chaperone for a macromolecular transcription-processing complex
Source: Nat Struct Mol Biol. 2025 Sep 4;32(11):2349–58. doi: 10.1038/s41594-025-01653-y (PMC12618233; doi:10.1038/s41594-025-01653-y)

Fig.1B

Core vRNAP with tRNA$^{Gln/Arg}$_ 1

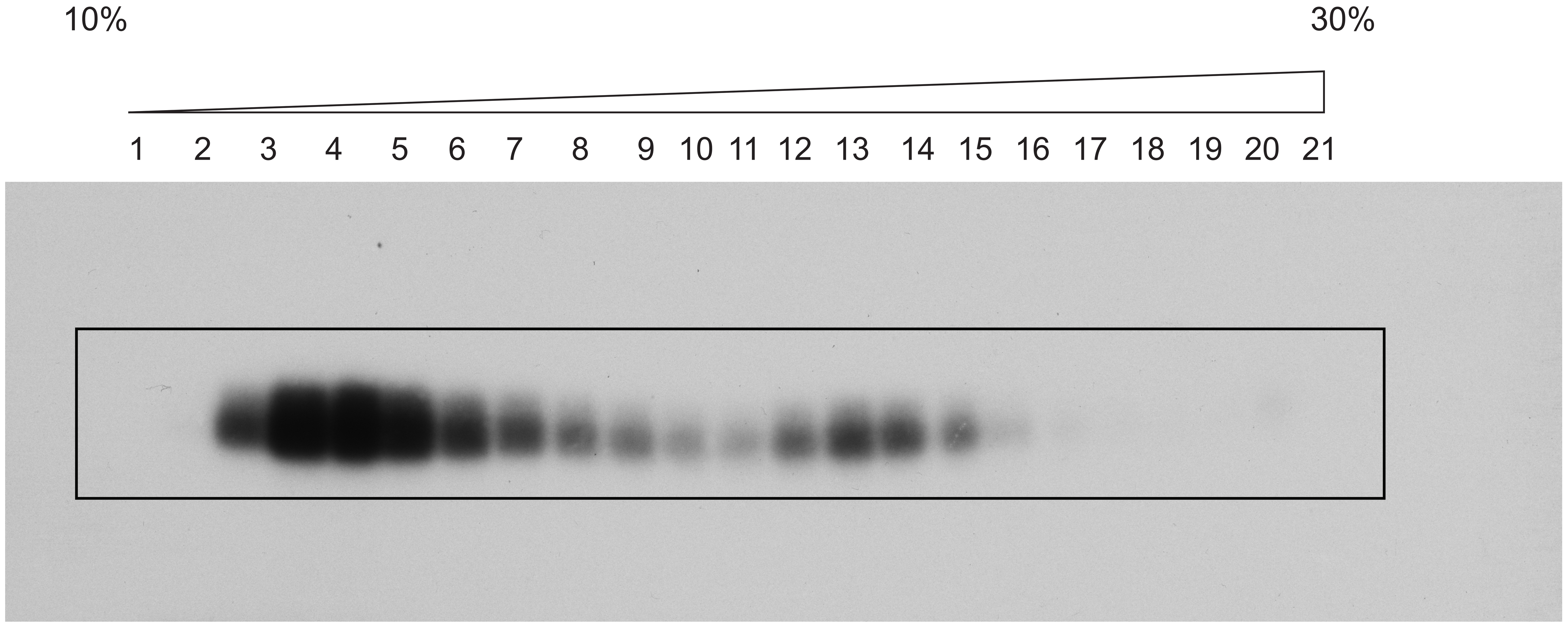

Core vRNAP with tRNA$^{Gln/Arg}$_2

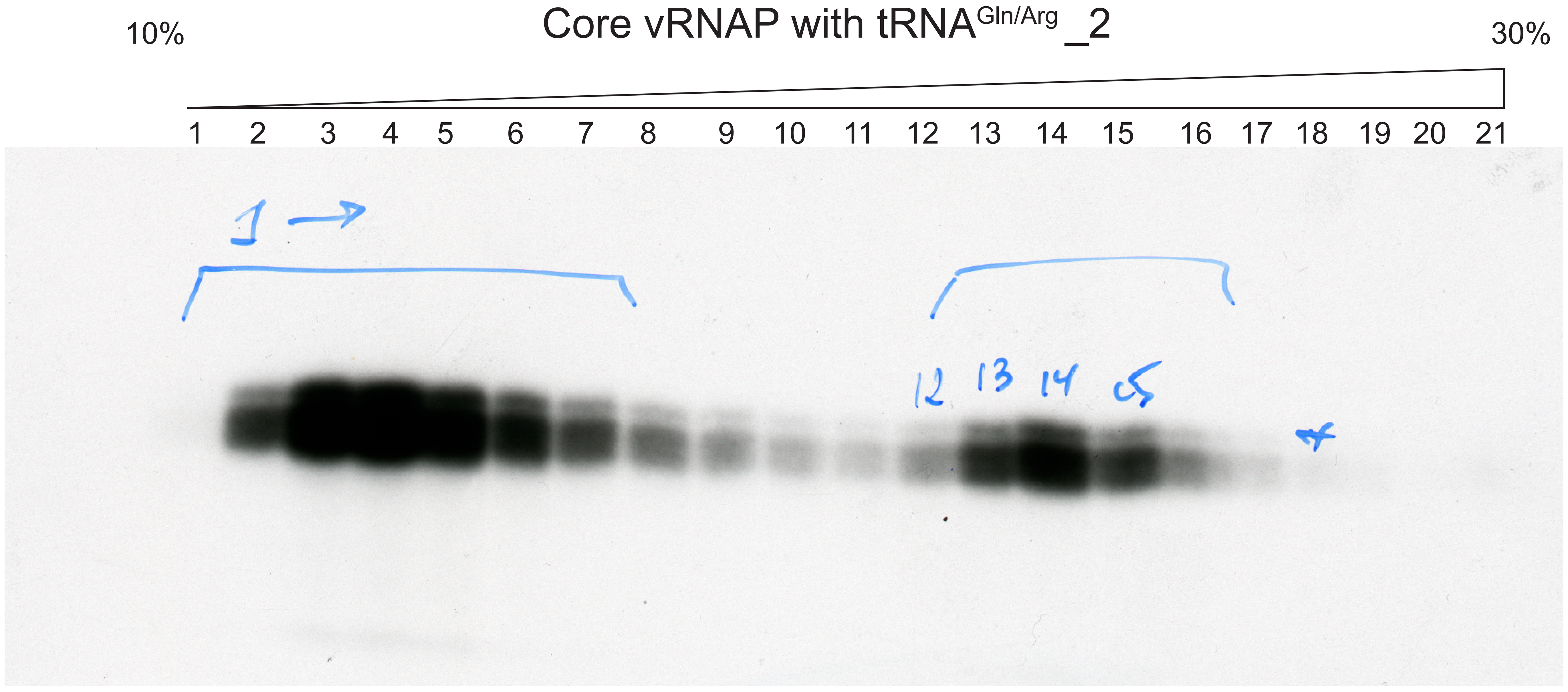

Core vRNAP with tRNA$^{Gln/Arg}$_3

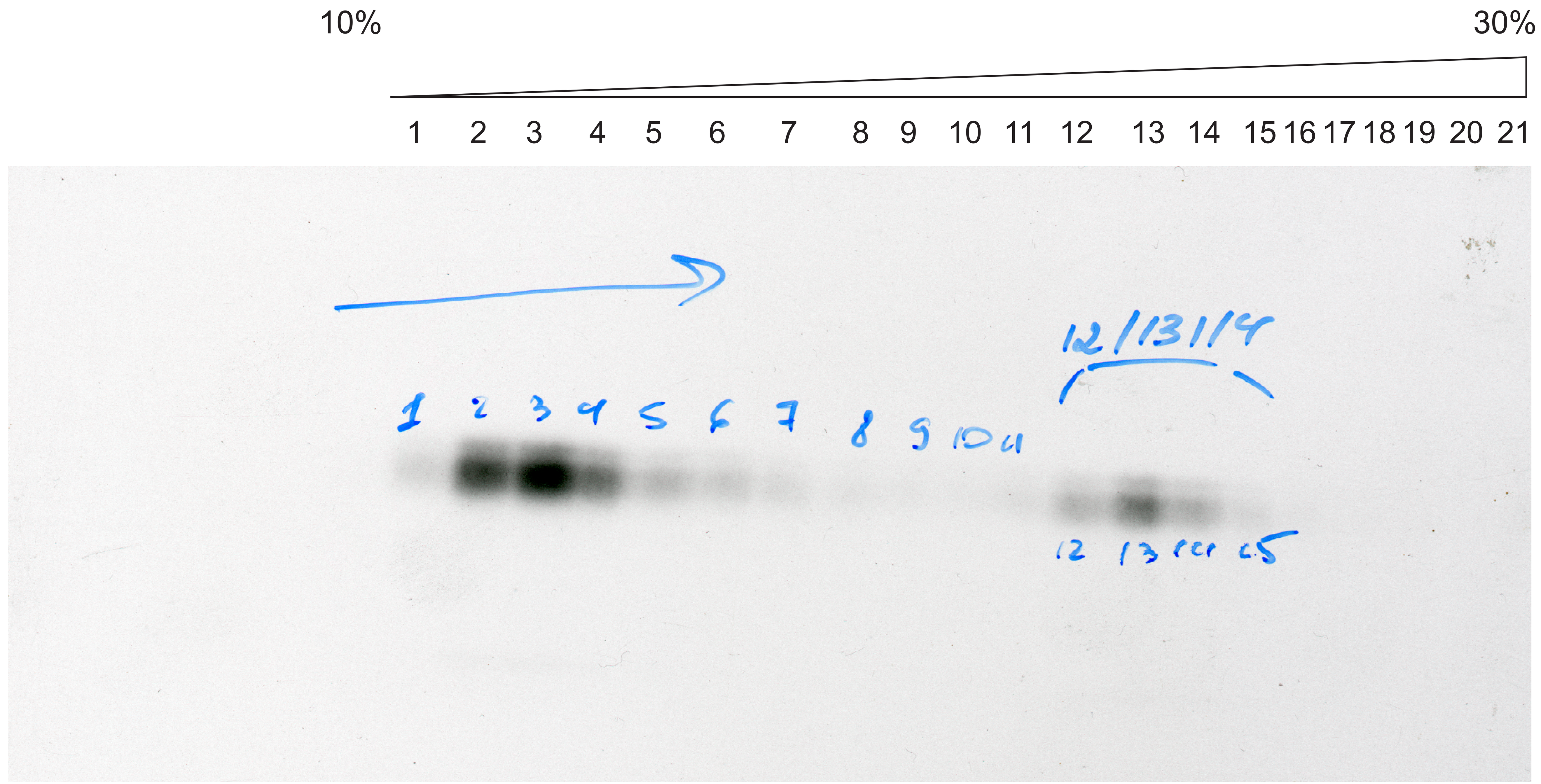

Fig.1B

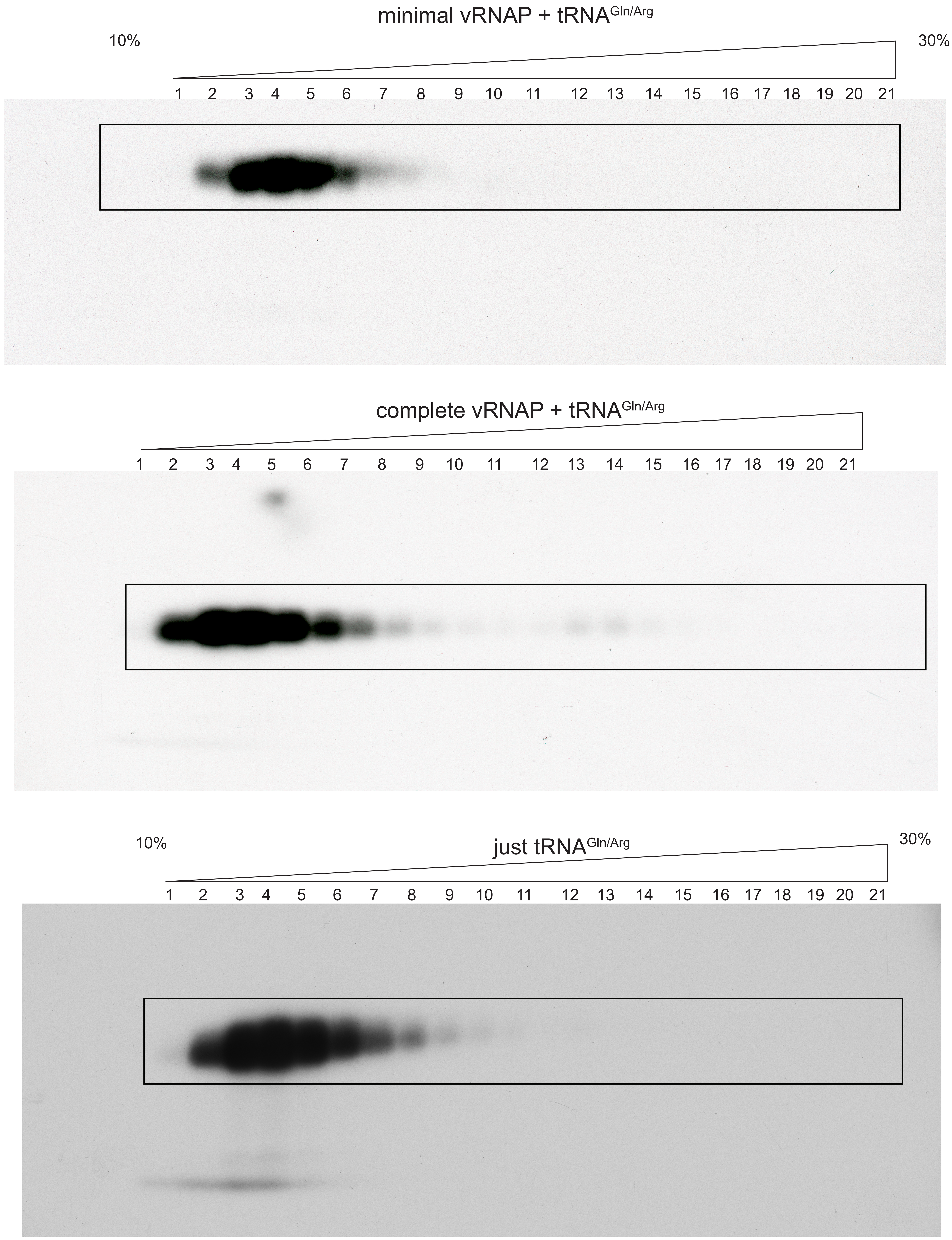
minimal vRNAP + tRNA^Gln/Arg
10%
30%
1 2 3 4 5 6 7 8 9 10 11 12 13 14 15 16 17 18 19 20 21
complete vRNAP + tRNA^Gln/Arg
1 2 3 4 5 6 7 8 9 10 11 12 13 14 15 16 17 18 19 20 21
10%
just tRNA^Gln/Arg
30%
1 2 3 4 5 6 7 8 9 10 11 12 13 14 15 16 17 18 19 20 21

Fig.1C

Shift assay_Core vRNAP/tRNA$^{Gln/Arg}$/E11/CE/NPH-I

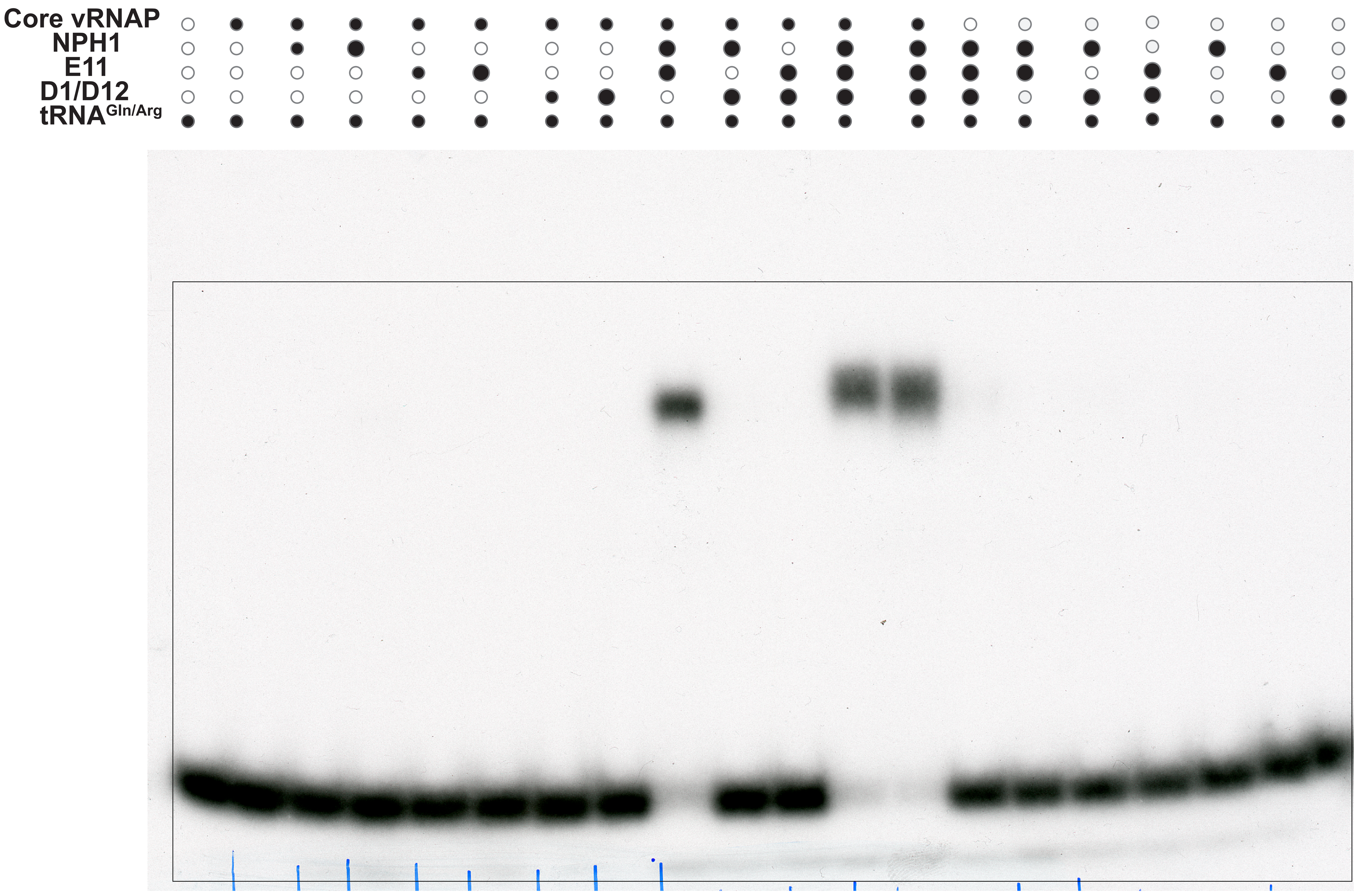

Shift assay_Core vRNAP/tRNA$^{Gln/Arg}$/E11/CE/NPH-I_2

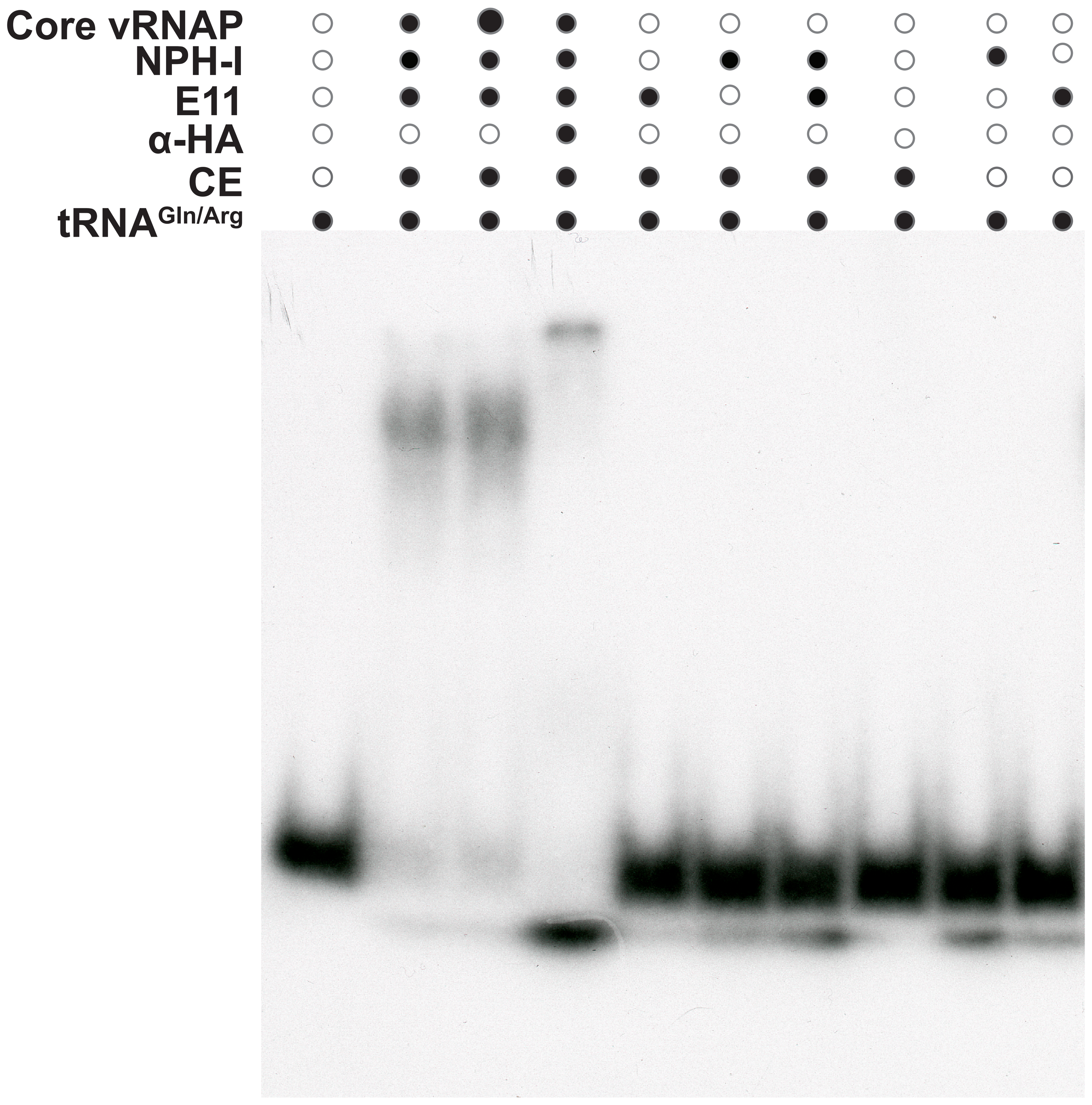

Shift assay_Core vRNAP/tRNA$^{Gln/Arg}$/E11/CE/NPH-I_3

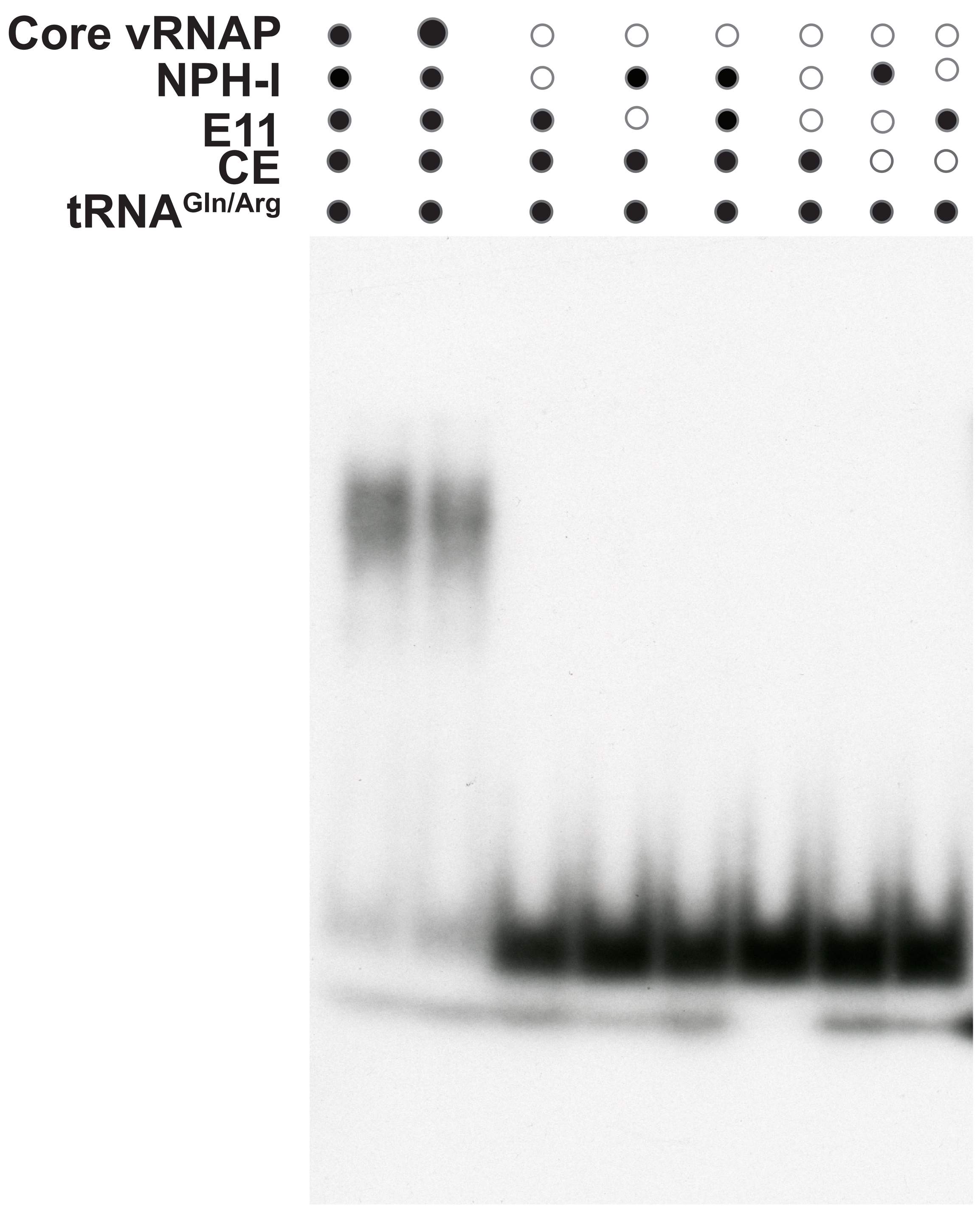

Shift assay_Core vRNAP/tRNA$^{Gln/Arg}$/E11/CE/NPH-I_4

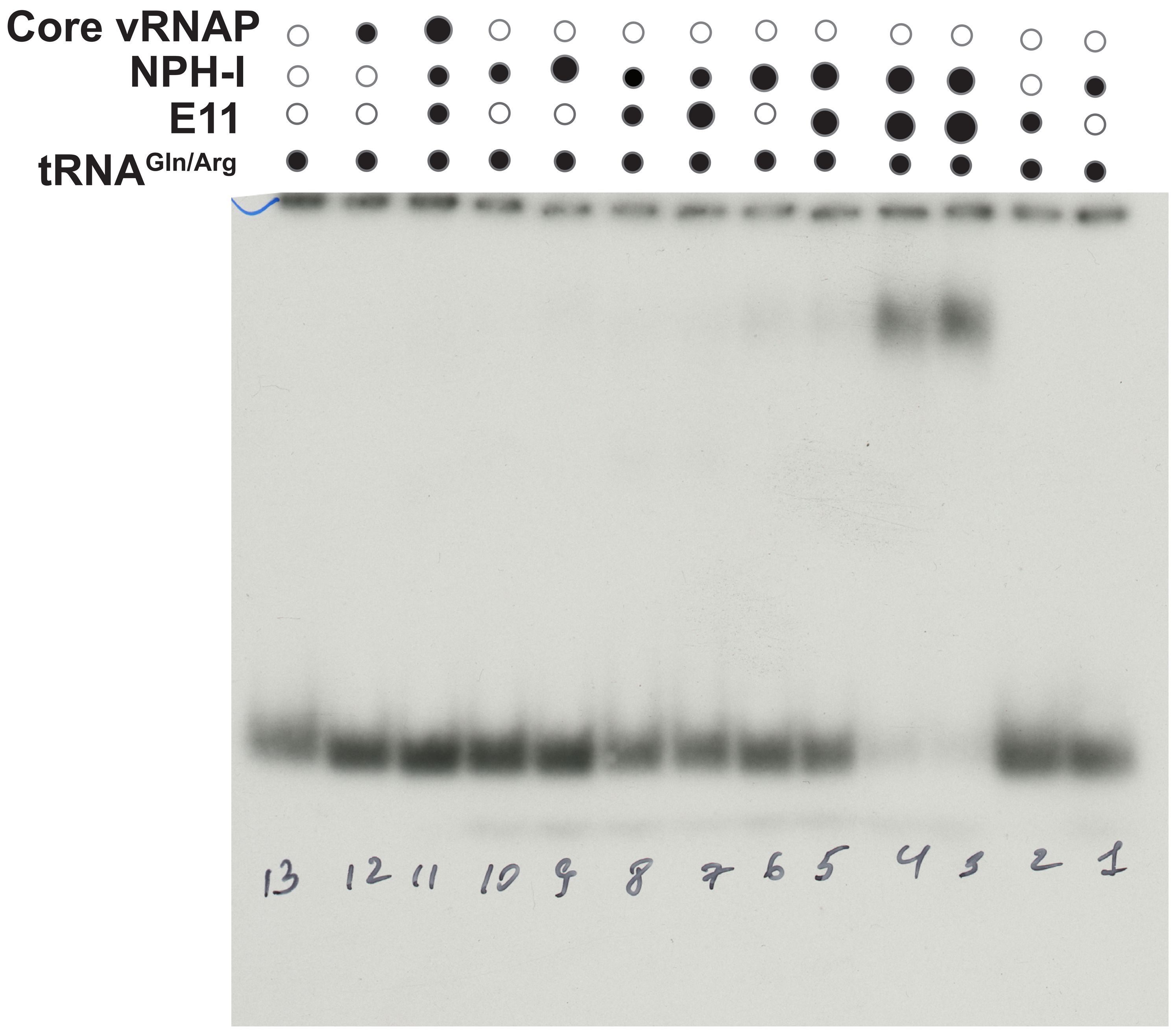

Supplement: Supplementary file 8 — Unprocessed X-ray films. [file 41594_2025_1653_MOESM8_ESM.pdf]
