# Supplementary material for: tRNA as an assembly chaperone for a macromolecular transcription-processing complex
Source: Nat Struct Mol Biol. 2025 Sep 4;32(11):2349–58. doi: 10.1038/s41594-025-01653-y (PMC12618233; doi:10.1038/s41594-025-01653-y)

Fig. 6A

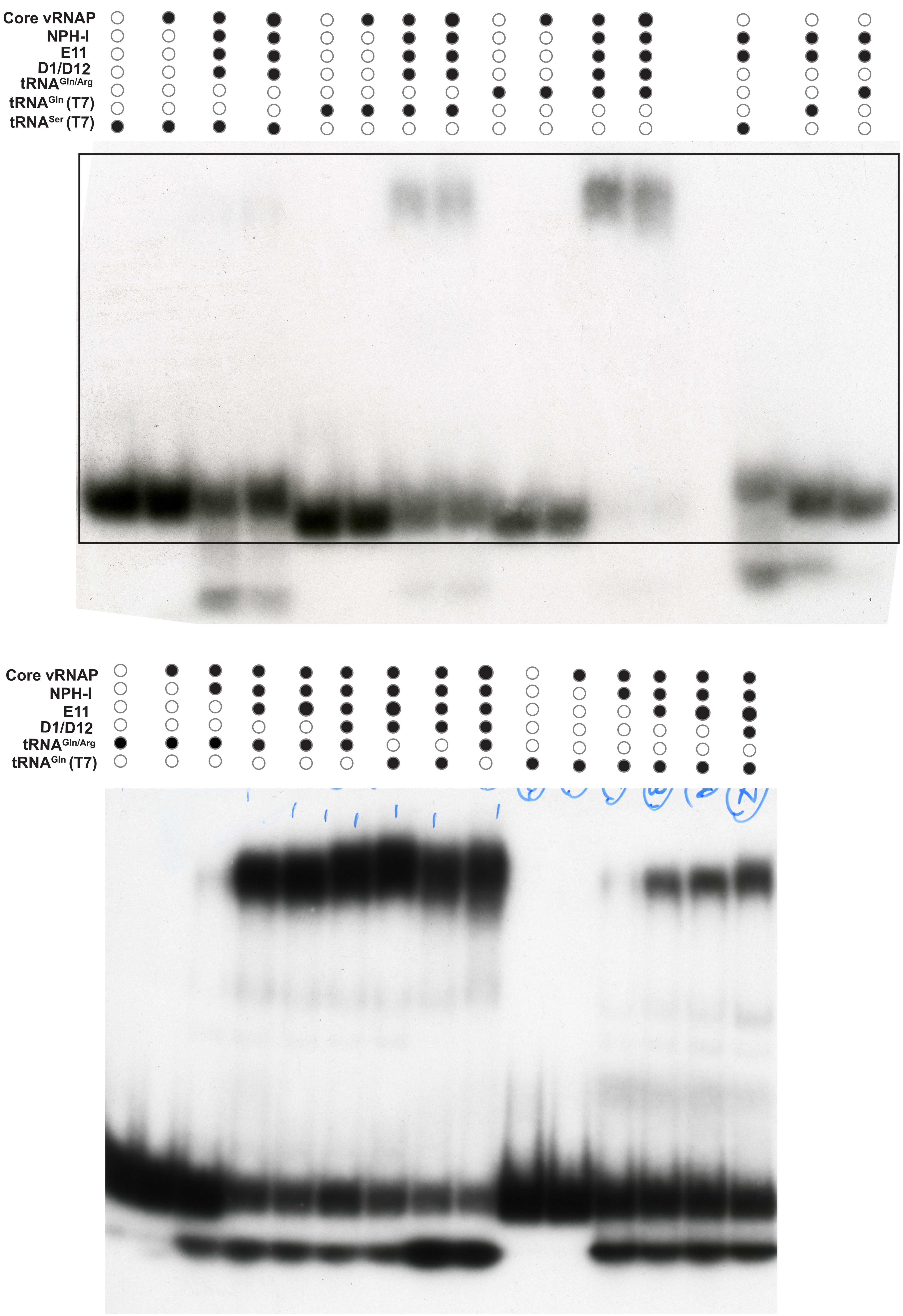
Core vRNAP
NPH-I
E11
D1/D12
tRNA Gln/Arg
tRNA Gln (T7)
tRNA Ser (T7)
Core vRNAP
NPH-I
E11
D1/D12
tRNA Gln/Arg
tRNA Gln (T7)

Fig.6E

Digestion of pCp-labeled tRNA$^{Gln/Arg}$, tryzolazed from complete vRNAP and total calf tRNAs by γ-Toxin

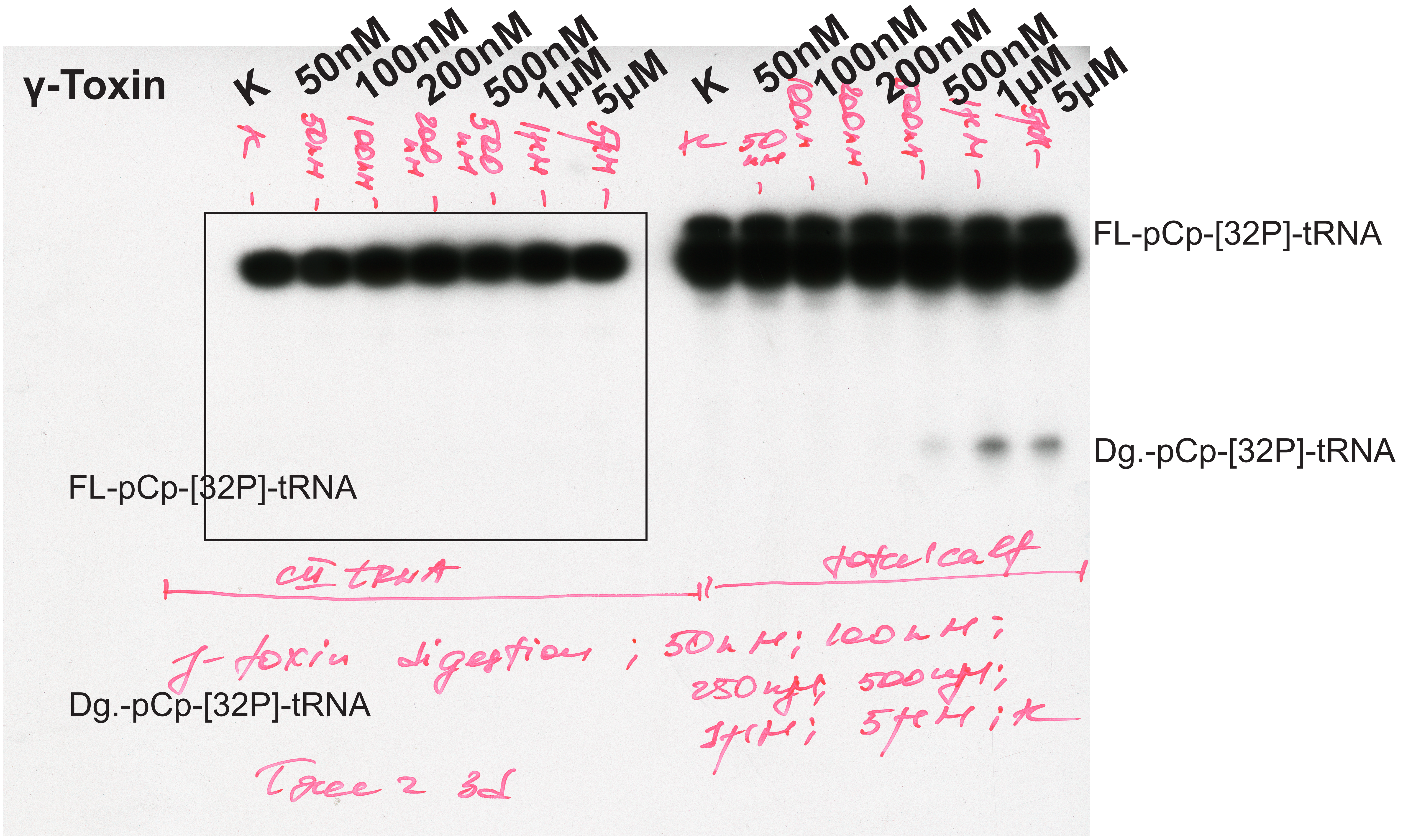

NB analysis of γ-Toxin digestion of tRNA^{Gln}(UUG) from vaccinia virus infected Hela S3 cells

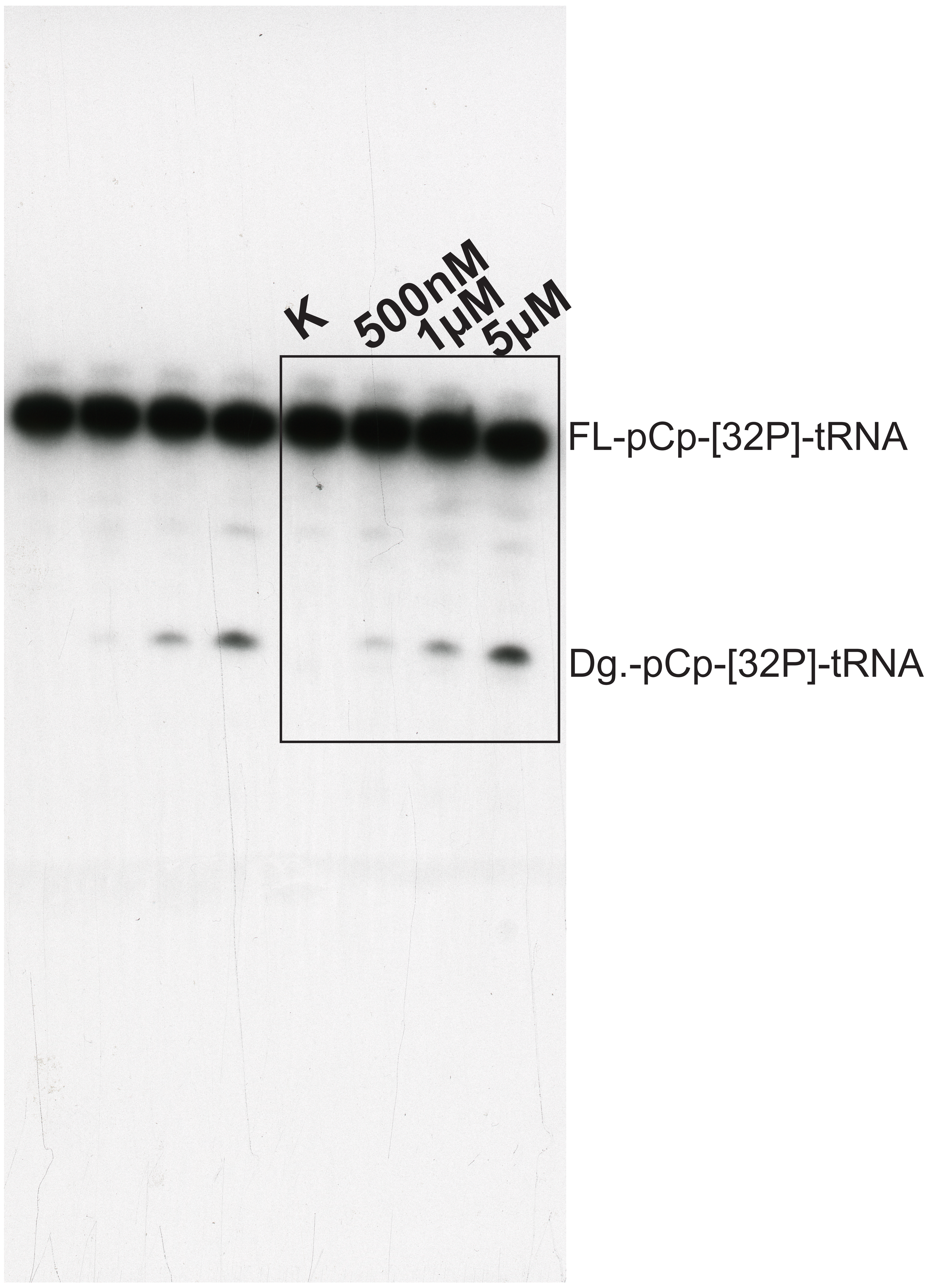

Supplement: Supplementary file 9 — Unprocessed X-ray films. [file 41594_2025_1653_MOESM9_ESM.pdf]
