# Supplementary material for: tRNA as an assembly chaperone for a macromolecular transcription-processing complex
Source: Nat Struct Mol Biol. 2025 Sep 4;32(11):2349–58. doi: 10.1038/s41594-025-01653-y (PMC12618233; doi:10.1038/s41594-025-01653-y)

Fig.8C

Nothern Blot analysis of MPVX vs VACV for tRNA^{Gln} (UUG)

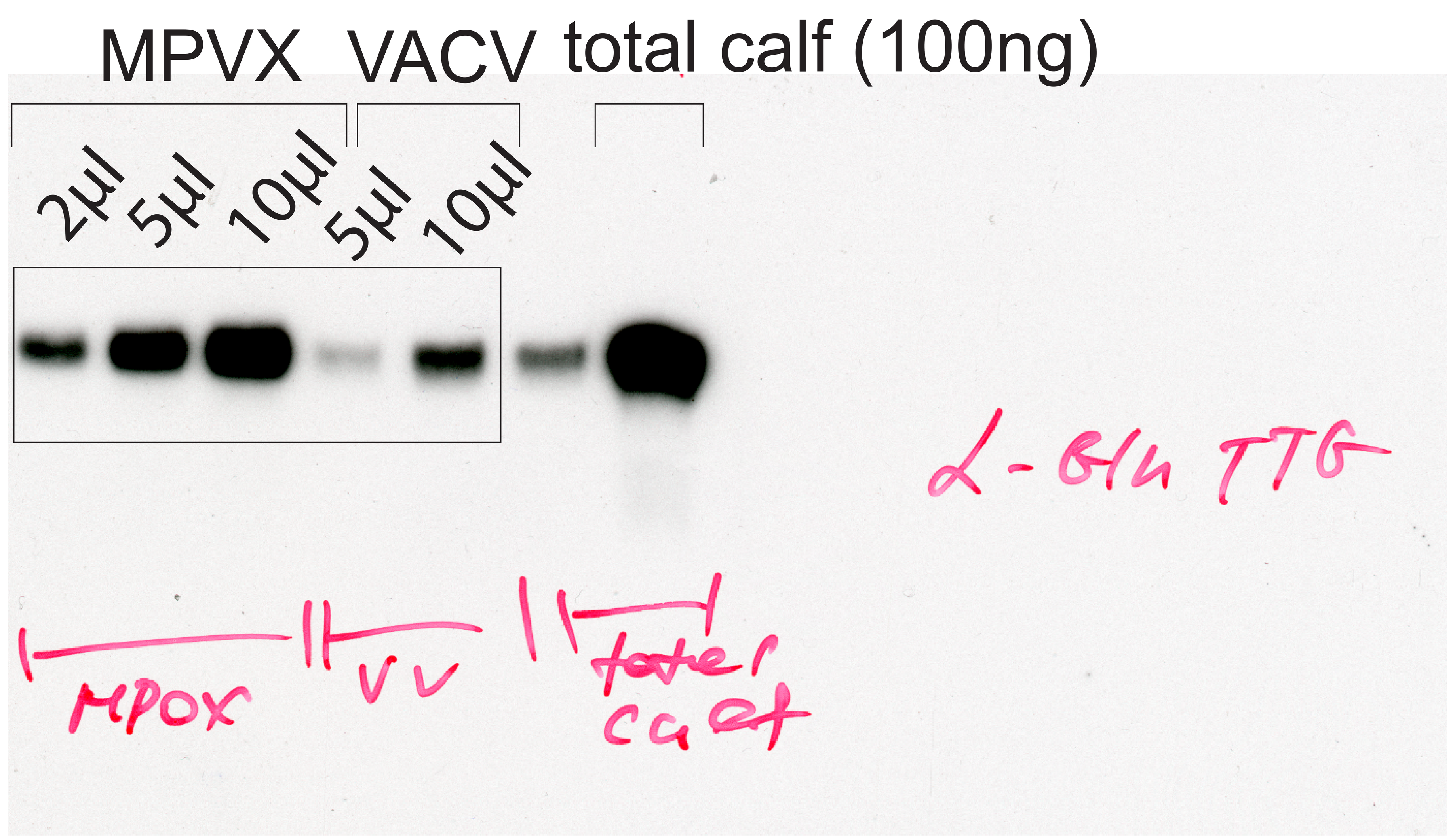

Nothern Blot analysis of MPVX vs VACV for tRNA^{Ser} (CGA)

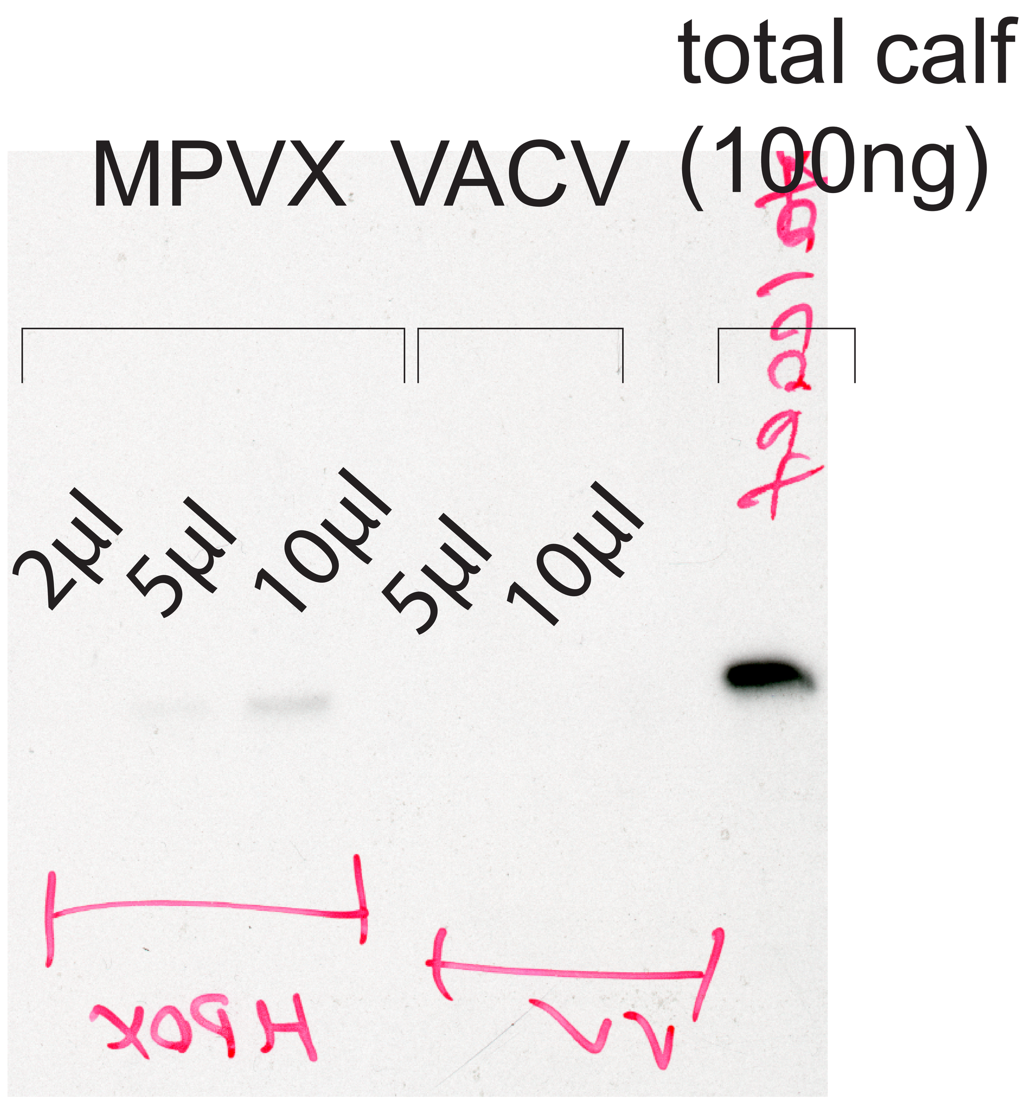

Nothern Blot analysis of MPVX vs VACV for tRNA^{arg} (CCG)

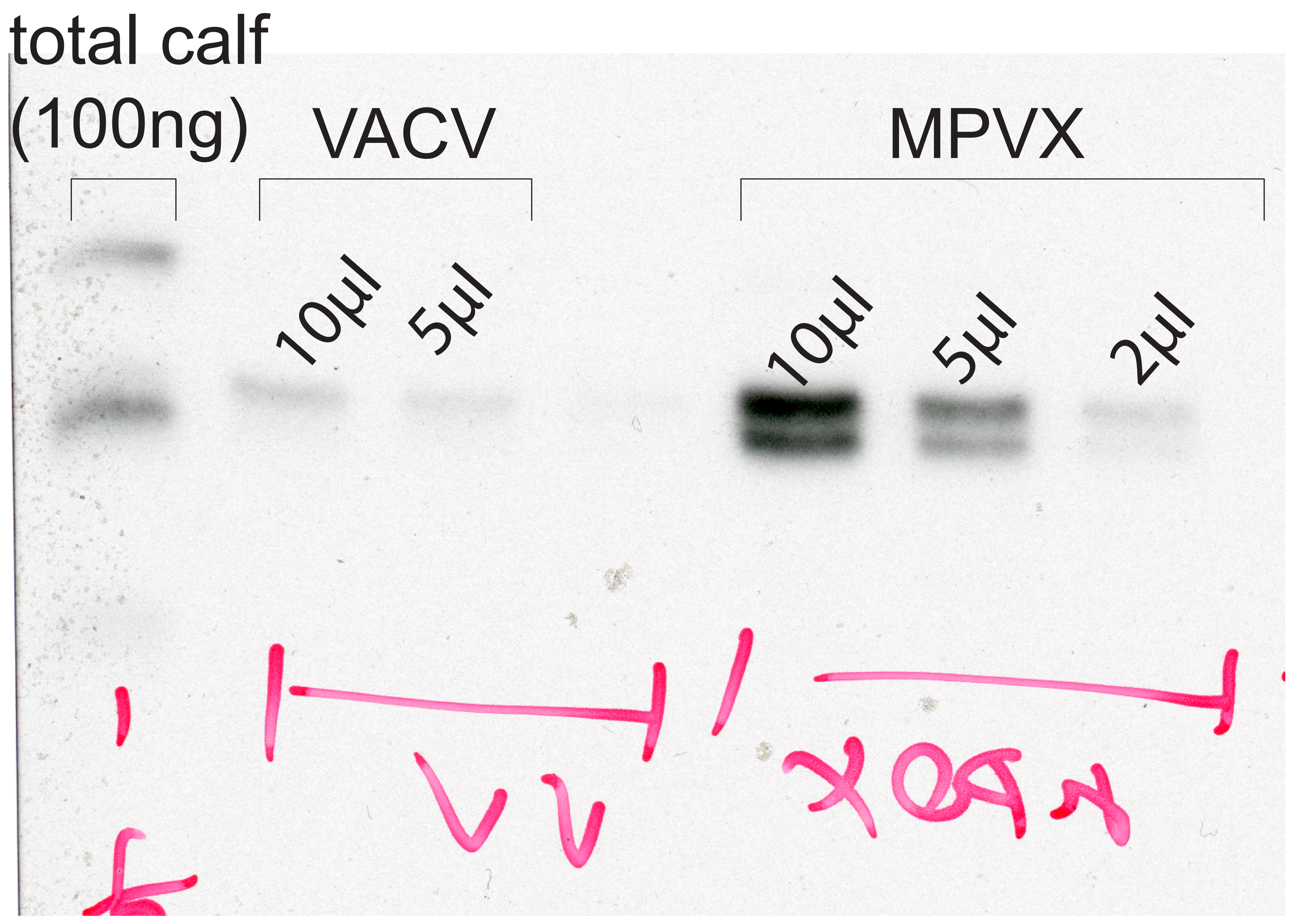

Supplement: Supplementary file 10 — Unprocessed X-ray films. [file 41594_2025_1653_MOESM10_ESM.pdf]
