# Supplementary material for: tRNA as an assembly chaperone for a macromolecular transcription-processing complex
Source: Nat Struct Mol Biol. 2025 Sep 4;32(11):2349–58. doi: 10.1038/s41594-025-01653-y (PMC12618233; doi:10.1038/s41594-025-01653-y)

Extended Data Fig.1A

CE E.coli Expression (Superdex 200)

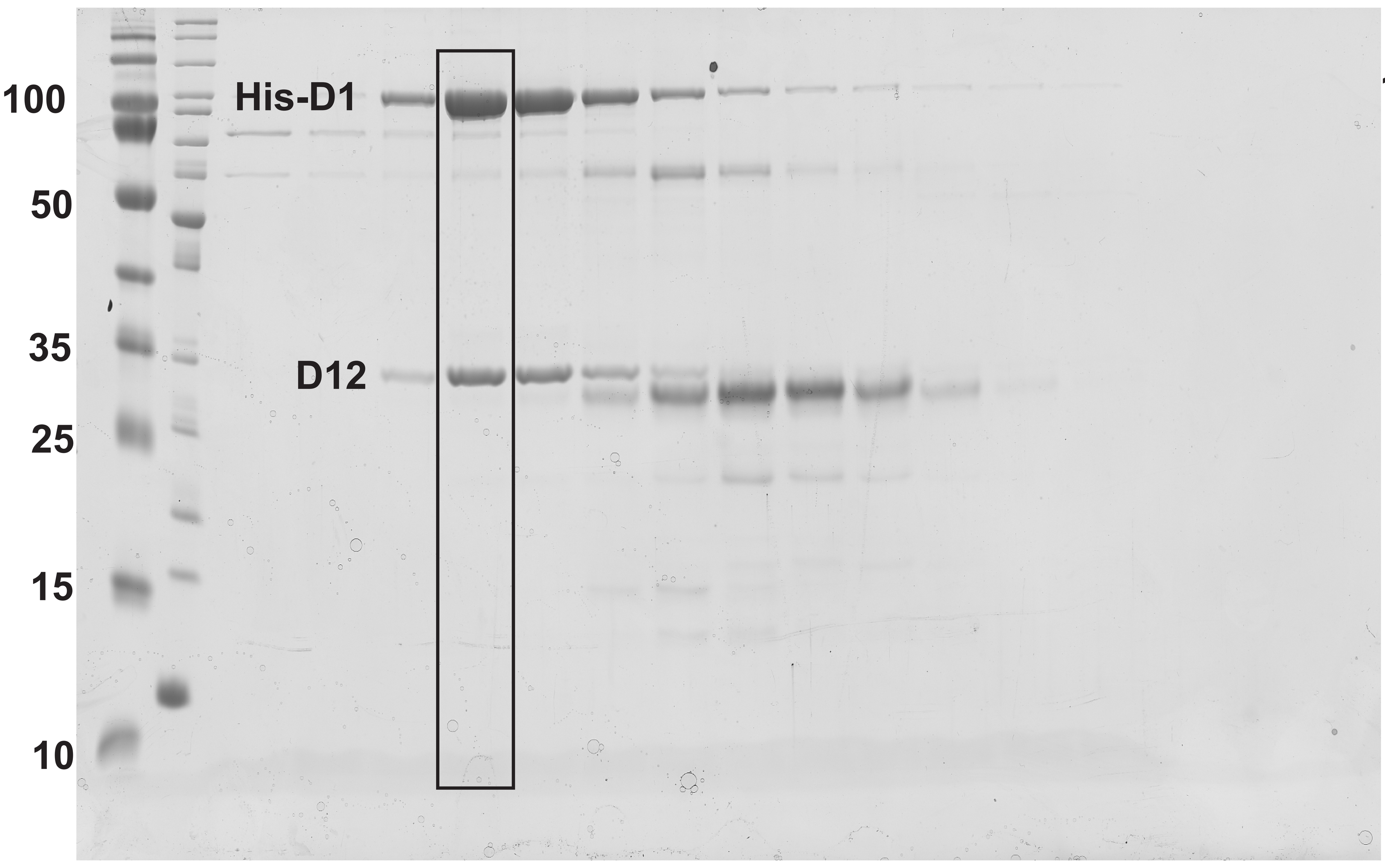

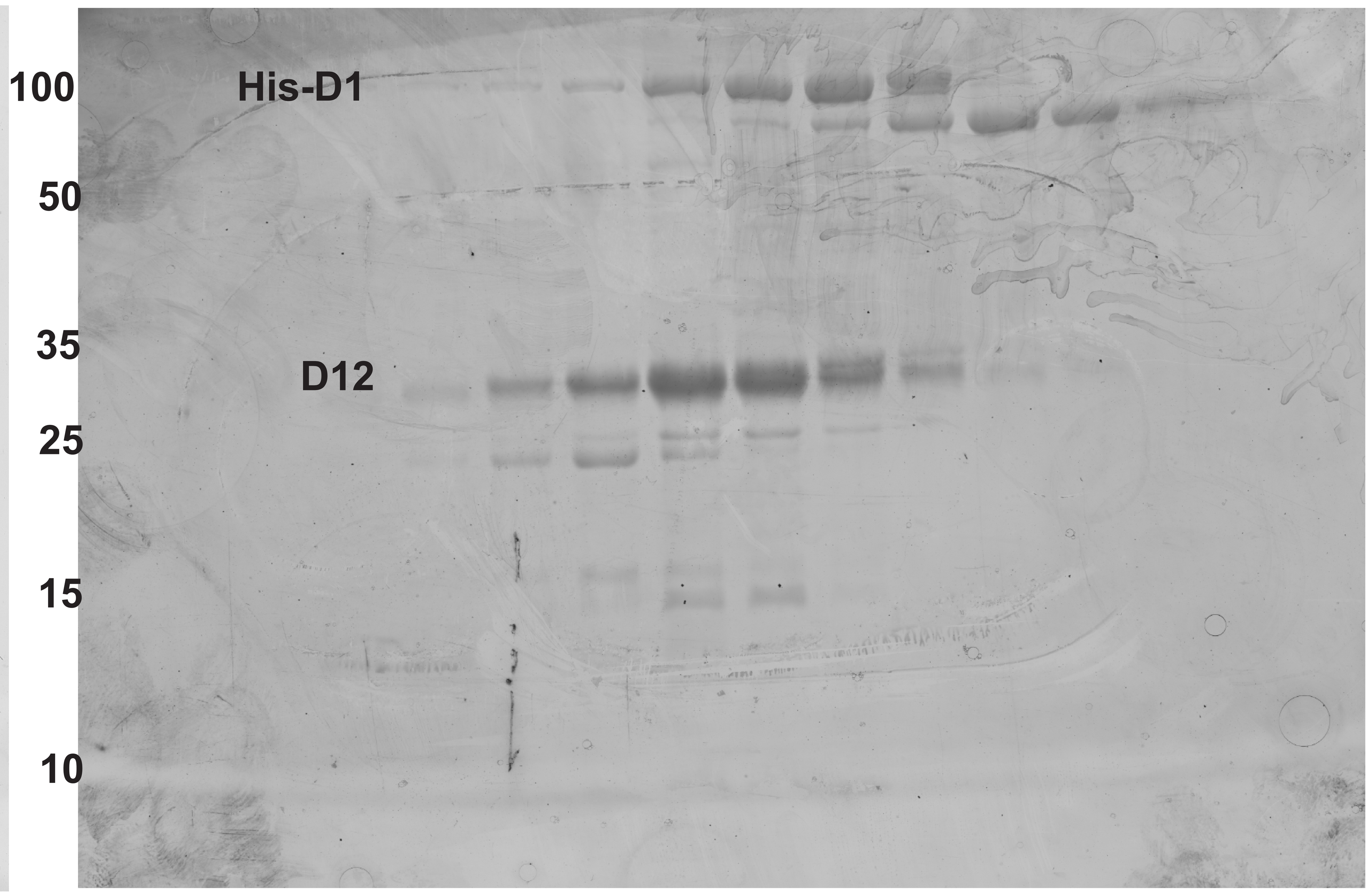

NPH-I E.coli Expression (Superdex 200)

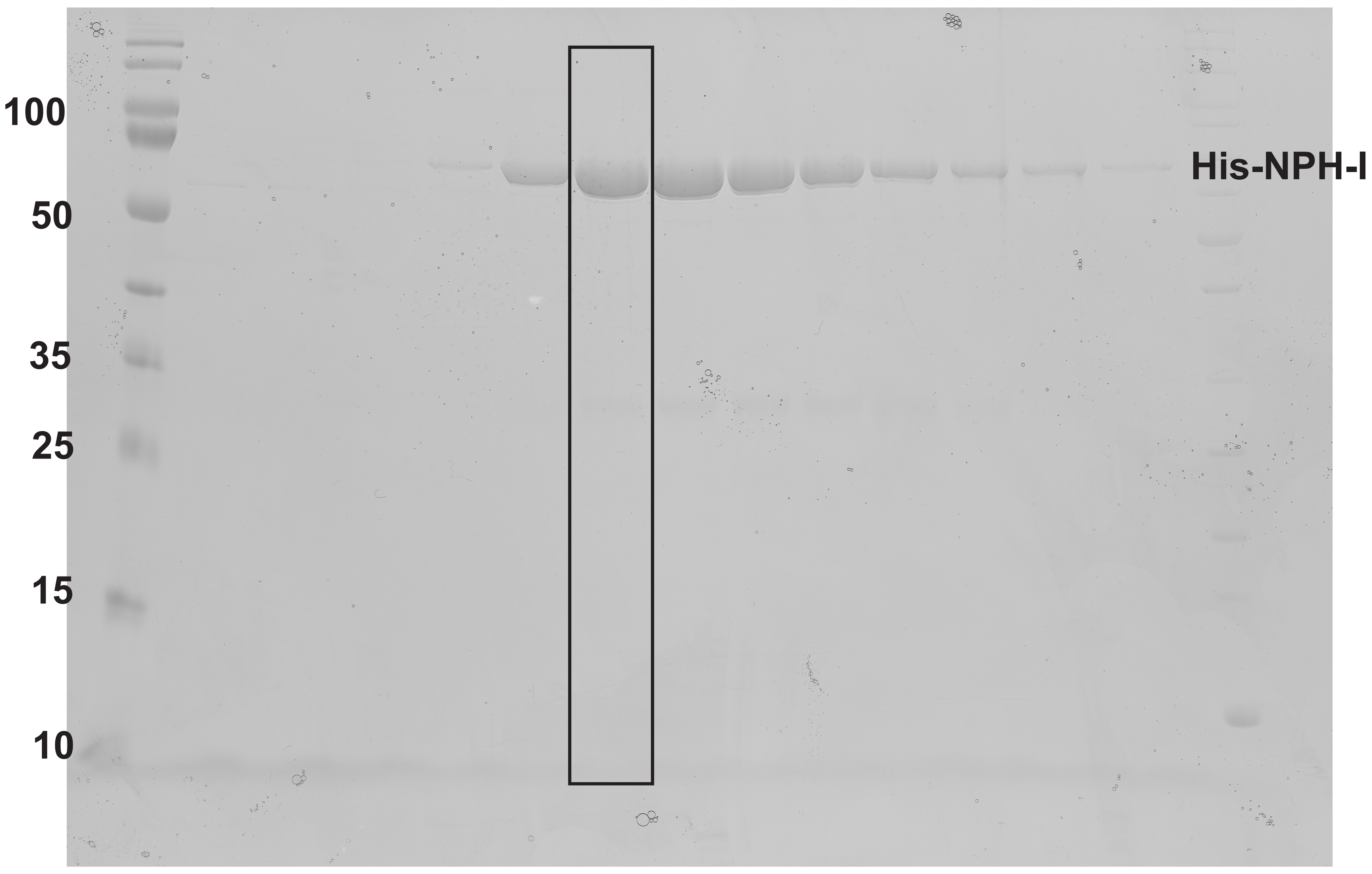

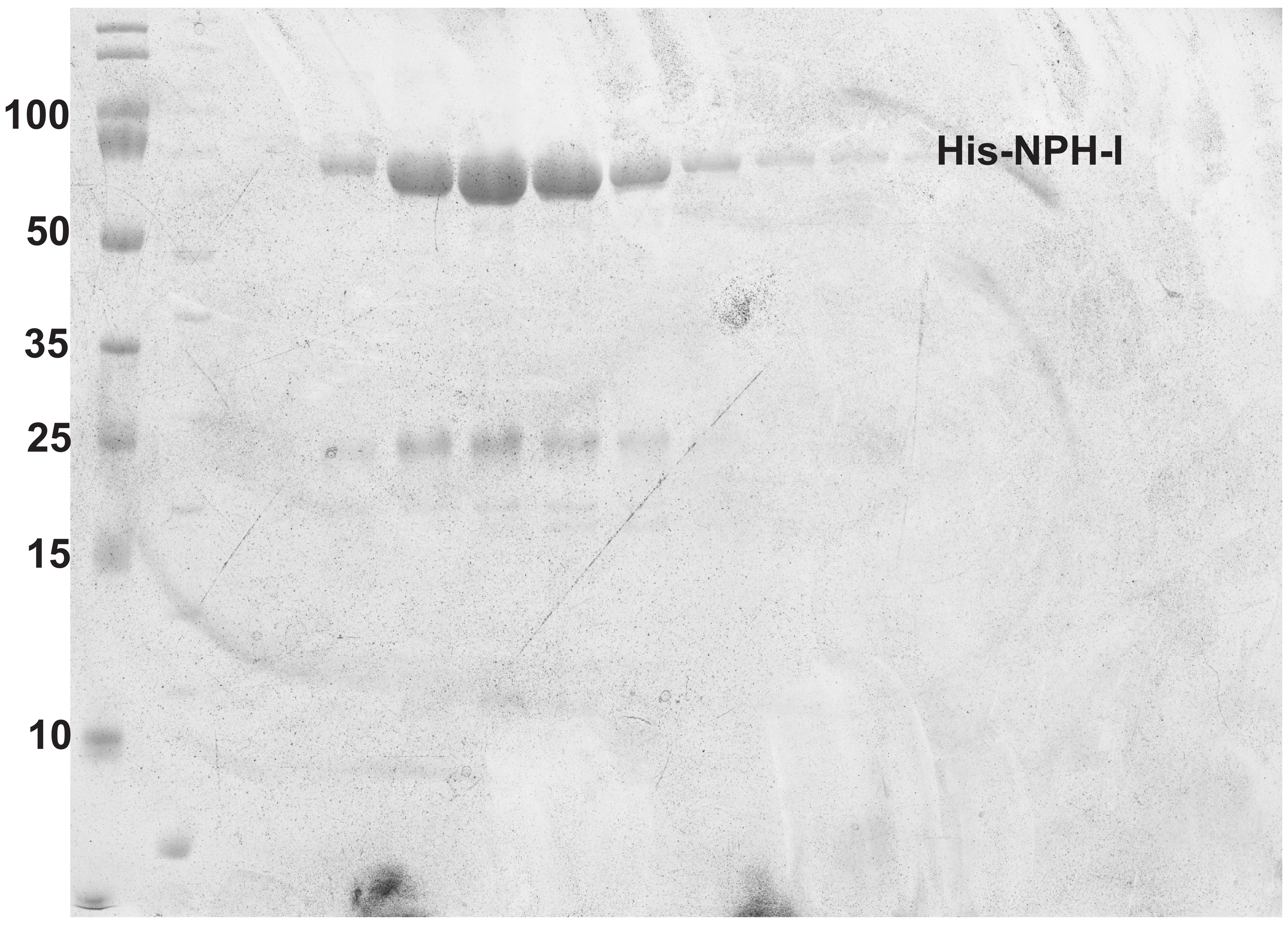

GST-γ-Toxin E.coli Expression (Superdex 200)

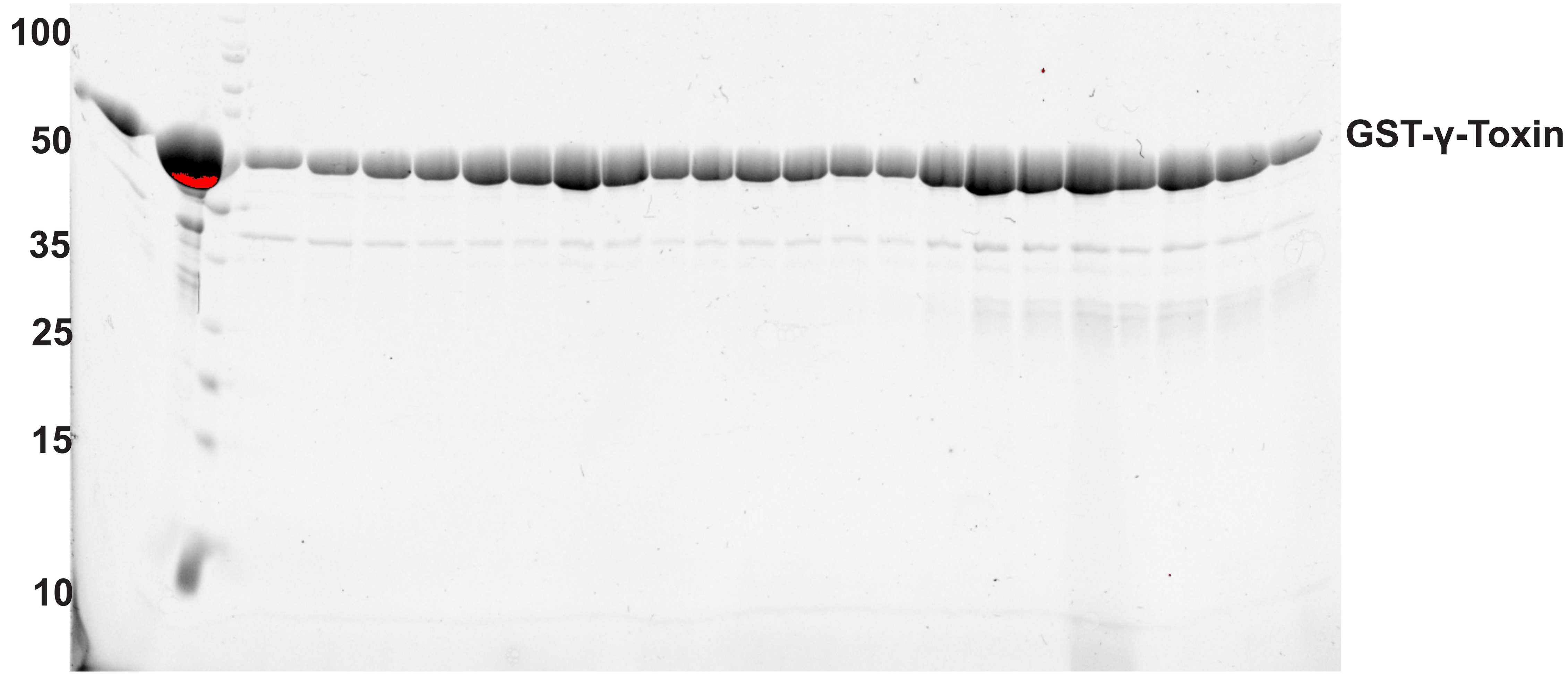

Extended Data Fig.1B

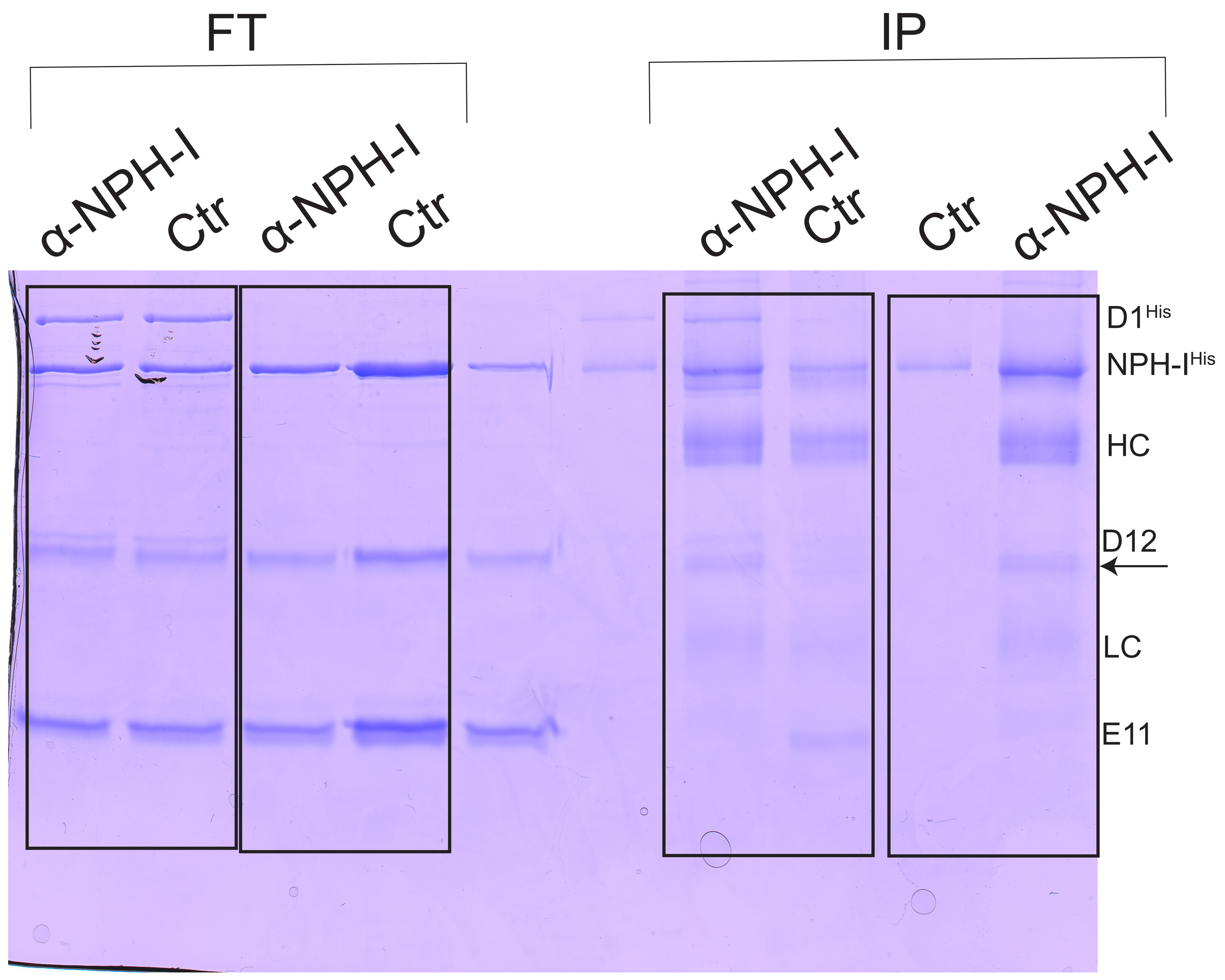

Extended Data Fig.1C

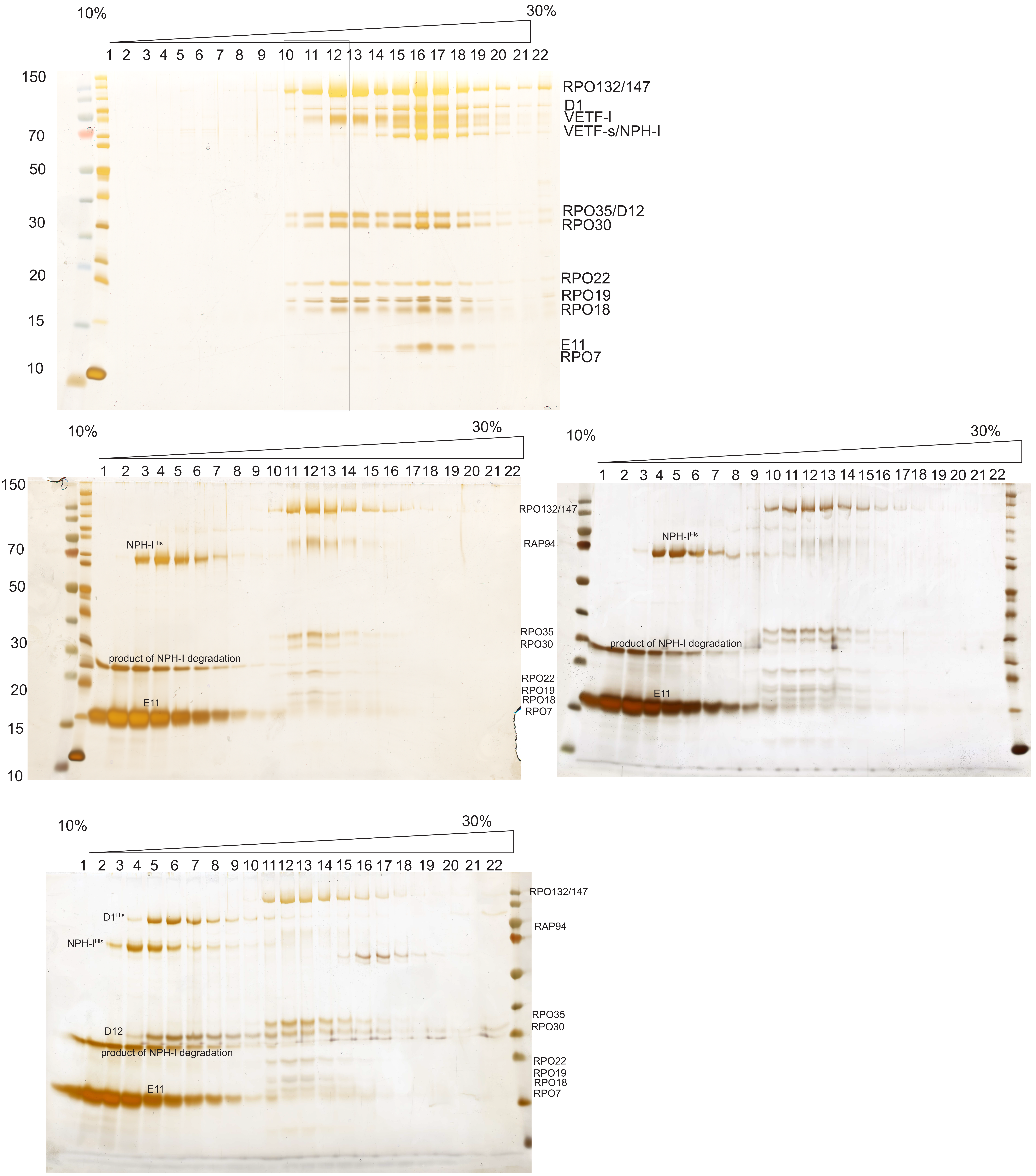

Supplement: Supplementary file 11 — Unprocessed gels. [file 41594_2025_1653_MOESM11_ESM.pdf]
