# Supplementary figures and images for: tRNA as an assembly chaperone for a macromolecular transcription-processing complex
Source: Nat Struct Mol Biol. 2025 Sep 4;32(11):2349–58. doi: 10.1038/s41594-025-01653-y (PMC12618233; doi:10.1038/s41594-025-01653-y)

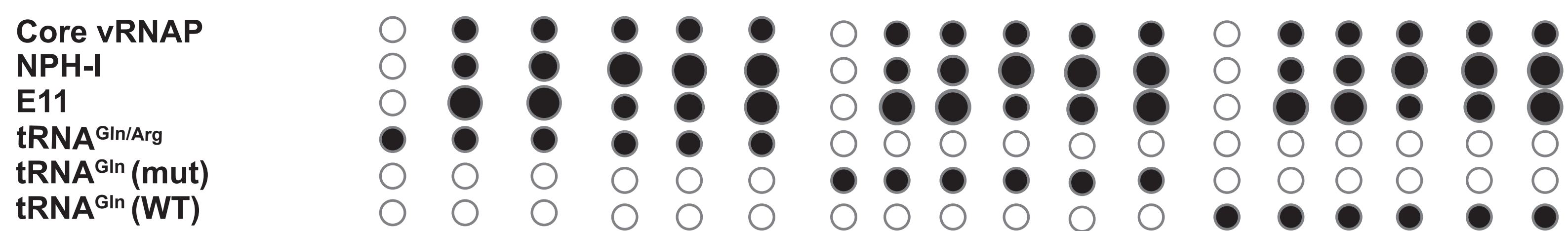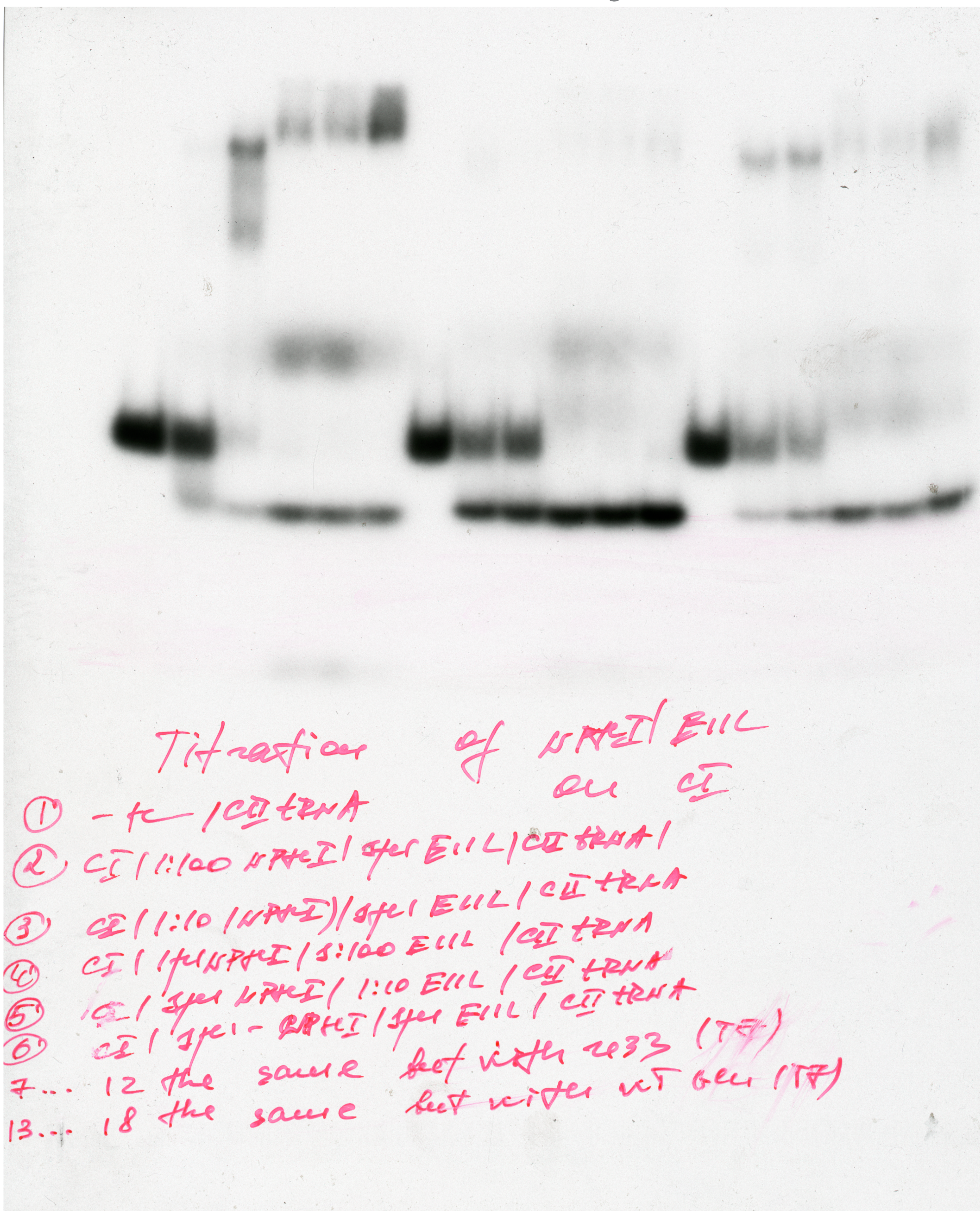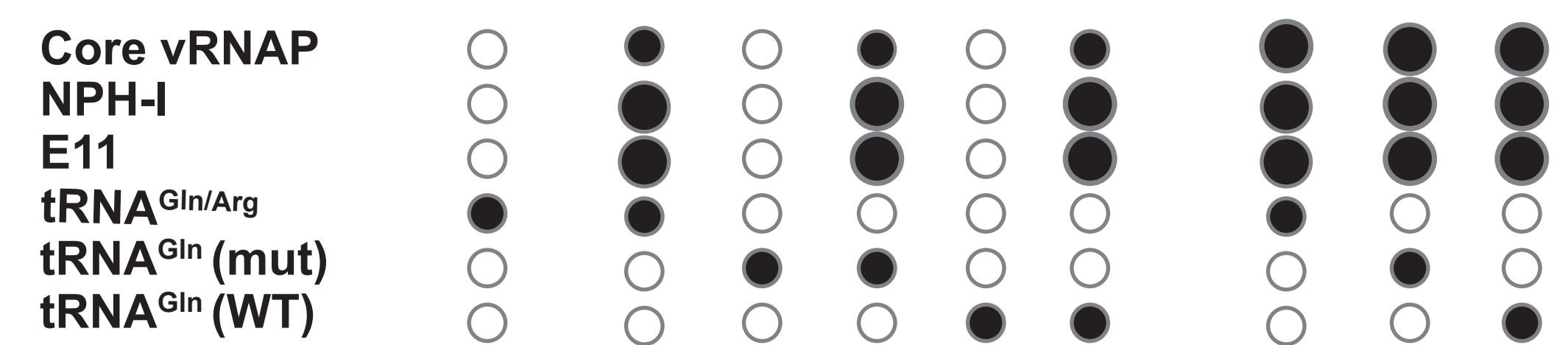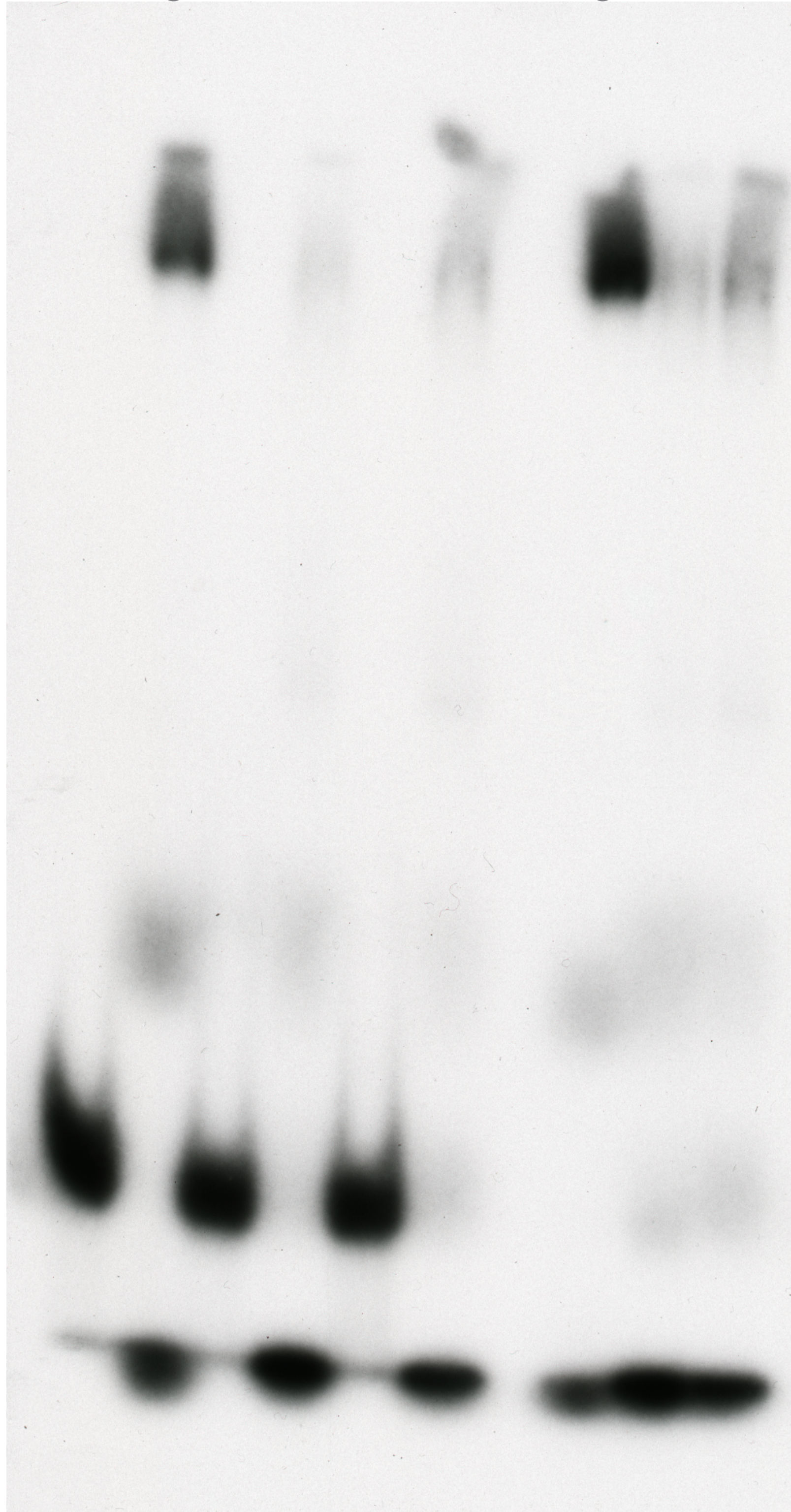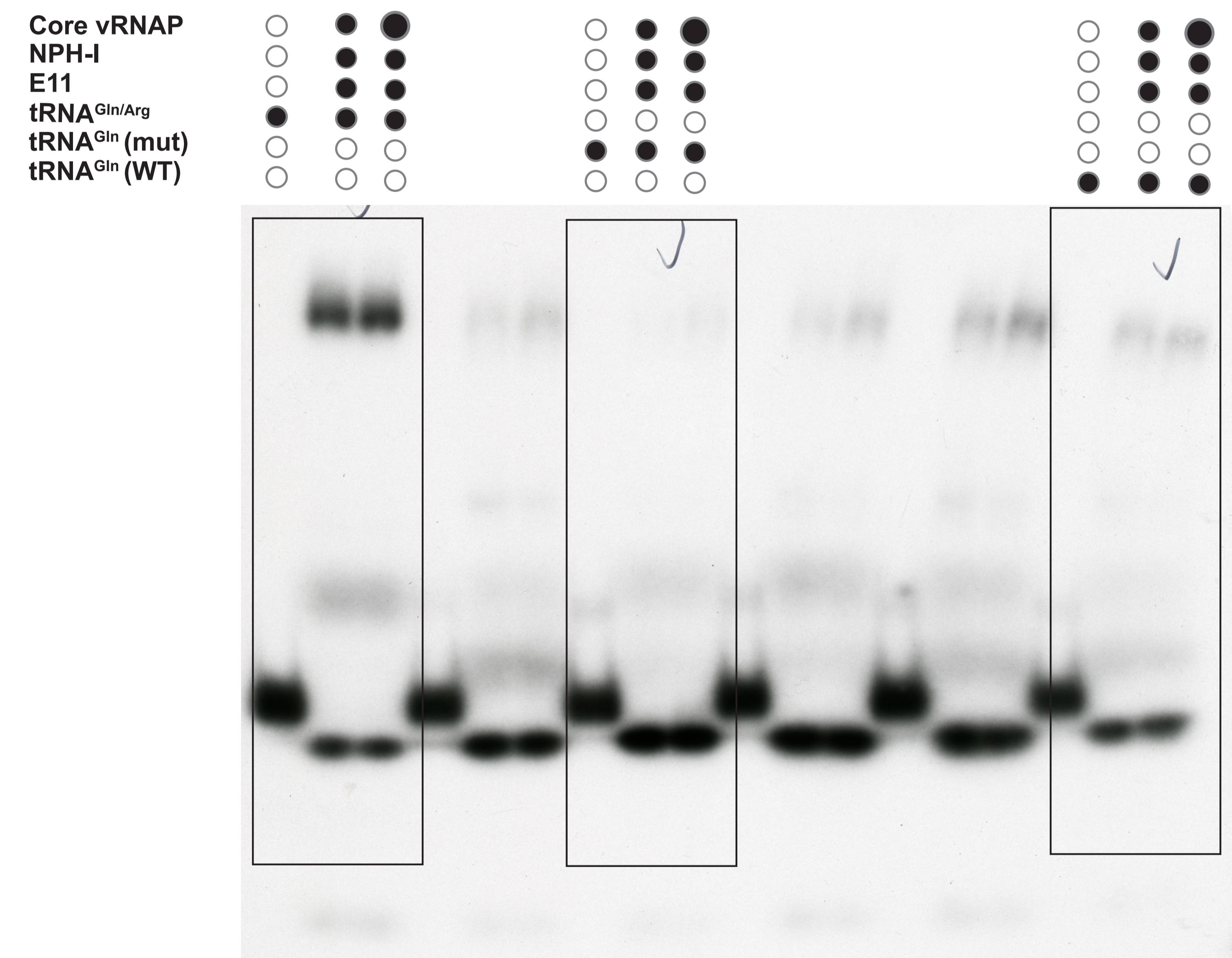

Supplement: Supplementary file 12 — Unprocessed X-ray films. [file 41594_2025_1653_MOESM12_ESM.pdf]
