# Supplementary material for: tRNA as an assembly chaperone for a macromolecular transcription-processing complex
Source: Nat Struct Mol Biol. 2025 Sep 4;32(11):2349–58. doi: 10.1038/s41594-025-01653-y (PMC12618233; doi:10.1038/s41594-025-01653-y)

Extended Data Fig.9

NB analysis; estimation amout of tRNA^{Gln}(TTG) in virion/complete vRNAP

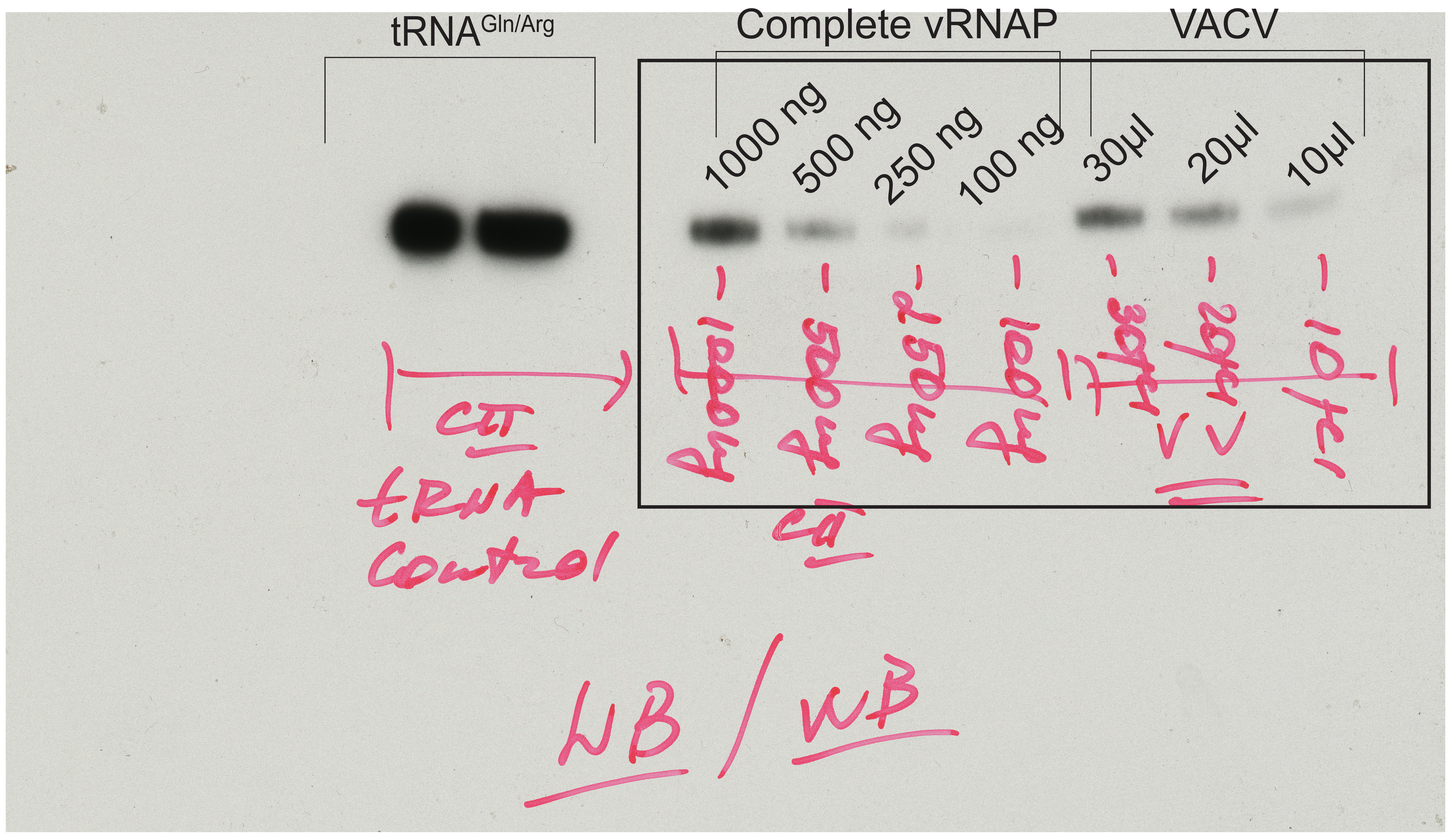

Anti-HA-RPO132

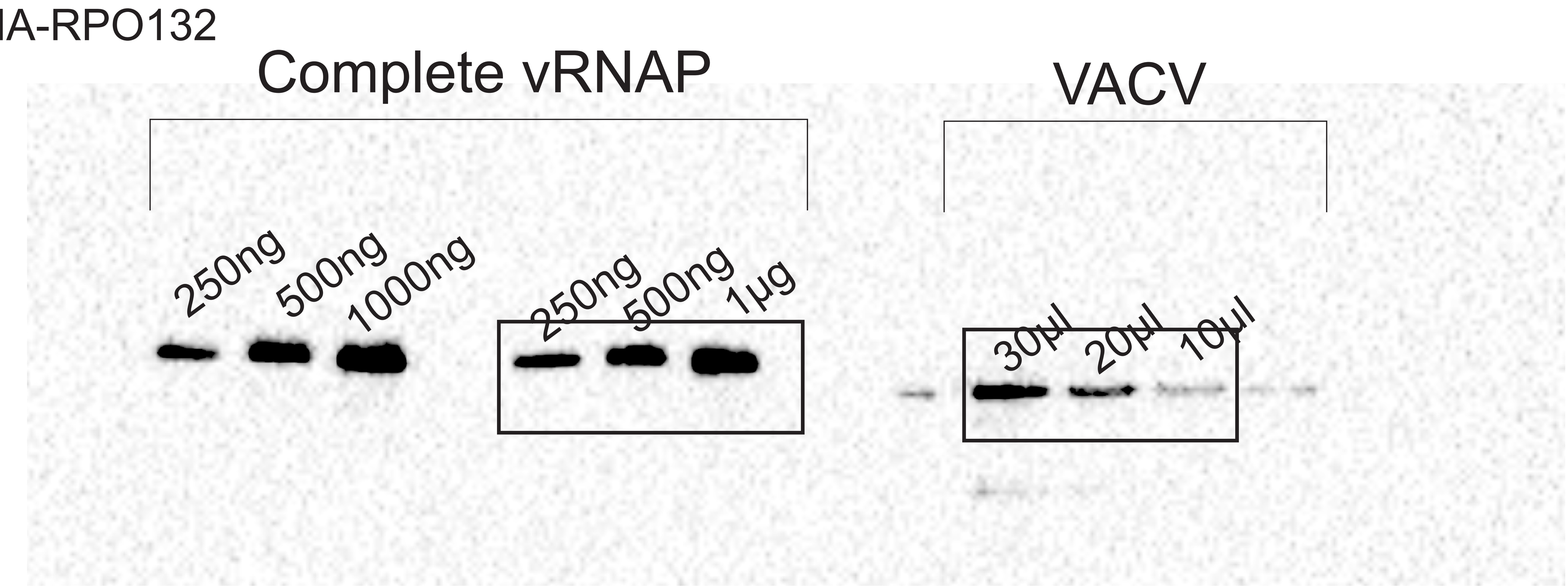

Supplement: Supplementary file 14 — Unprocessed X-ray films. [file 41594_2025_1653_MOESM14_ESM.pdf]
